# Single-cell profiling of myasthenia gravis identifies a pathogenic T cell signature

**DOI:** 10.1007/s00401-021-02299-y

**Published:** 2021-03-28

**Authors:** Florian Ingelfinger, Sinduya Krishnarajah, Michael Kramer, Sebastian G. Utz, Edoardo Galli, Mirjam Lutz, Pascale Zwicky, Ayse U. Akarca, Nicole Puertas Jurado, Can Ulutekin, David Bamert, Corinne C. Widmer, Luca Piccoli, Federica Sallusto, Nicolás G. Núñez, Teresa Marafioti, Didier Schneiter, Isabelle Opitz, Antonio Lanzavecchia, Hans H. Jung, Donatella De Feo, Sarah Mundt, Bettina Schreiner, Burkhard Becher

**Affiliations:** 1 Institute of Experimental Immunology, University of Zurich, Zurich, Switzerland; 2 Department of Neurology, University Hospital Zurich, Zurich, Switzerland; 3 Institute for Research in Biomedicine, Università Della Svizzera Italiana, Bellinzona, Switzerland; 4 Department of Cellular Pathology, University College London Hospital, London, UK; 5 Department of Medical Oncology and Hematology, University Hospital Zurich and University of Zurich, Zurich, Switzerland; 6 Institute of Microbiology, ETH Zurich, Zurich, Switzerland; 7 Department of Thoracic Surgery, University Hospital Zurich, Zurich, Switzerland

**Keywords:** Tissue-resident T cells, Biomarker, Autoimmunity, Mass cytometry, Immunophenotyping, Thymus, Myasthenia gravis, Cytokines

## Abstract

Myasthenia gravis (MG) is an autoimmune disease characterized by impaired neuromuscular signaling due to autoantibodies targeting the acetylcholine receptor. Although its auto-antigens and effector mechanisms are well defined, the cellular and molecular drivers underpinning MG remain elusive. Here, we employed high-dimensional single-cell mass and spectral cytometry of blood and thymus samples from MG patients in combination with supervised and unsupervised machine-learning tools to gain insight into the immune dysregulation underlying MG. By creating a comprehensive immune map, we identified two dysregulated subsets of inflammatory circulating memory T helper (Th) cells. These signature Th_CD103_ and Th_GM_ cells populated the diseased thymus, were reduced in the blood of MG patients, and were inversely correlated with disease severity. Both signature Th subsets rebounded in the blood of MG patients after surgical thymus removal, indicative of their role as cellular markers of disease activity. Together, this in-depth analysis of the immune landscape of MG provides valuable insight into disease pathogenesis, suggests novel biomarkers and identifies new potential therapeutic targets for treatment.

**Supplementary Information:**

The online version contains supplementary material available at 10.1007/s00401-021-02299-y.

## Introduction

Myasthenia gravis (MG) is an antibody-mediated autoimmune disease affecting neuromuscular transmission. Most MG patients have autoantibodies recognizing the acetylcholine receptor (AChR), leading to potentially life-threatening muscle weakness [1]. Despite their central role in MG pathology, AChR antibody levels do not correlate well with disease severity or treatment responses [37], highlighting our lack of knowledge of the underlying mechanisms of the disease. There is clear evidence of the key role of the thymus in inducing and maintaining MG [23], and several studies have implicated specific cell populations besides antibody-producing B cells, such as type 1 T helper (Th1) and Th17 cells [21], regulatory T cells (T_regs_) [2] and follicular Th cells (T_FH_) [47]. However, a comprehensive unbiased analysis of the immune cells involved in both the thymus and periphery of MG patients is lacking. Here, we employed an immunophenotyping approach to provide insight into the cellular and molecular dysregulation leading to autoimmunity in MG patients. By combining mass and spectral flow cytometry with data-driven machine-learning tools, we identified a pathogenic Th cell signature in MG that, unlike autoantibody levels, correlated closely with disease severity. Signature Th cell subsets accumulated in the MG thymus and were restored in the blood after thymectomy. Our study has identified a novel immune signature that upon validation using multicenter large-scale studies could be used as a biomarker to monitor disease severity and inform clinical management of MG patients. Moreover, these results provide a unifying analysis for further interrogation of the cellular mechanisms of MG, and offer the potential to identify novel therapeutic targets for treatment of this disease.

## Results

### Major blood immune cell populations are comparable in MG patients and healthy controls

**Fig. 1:**
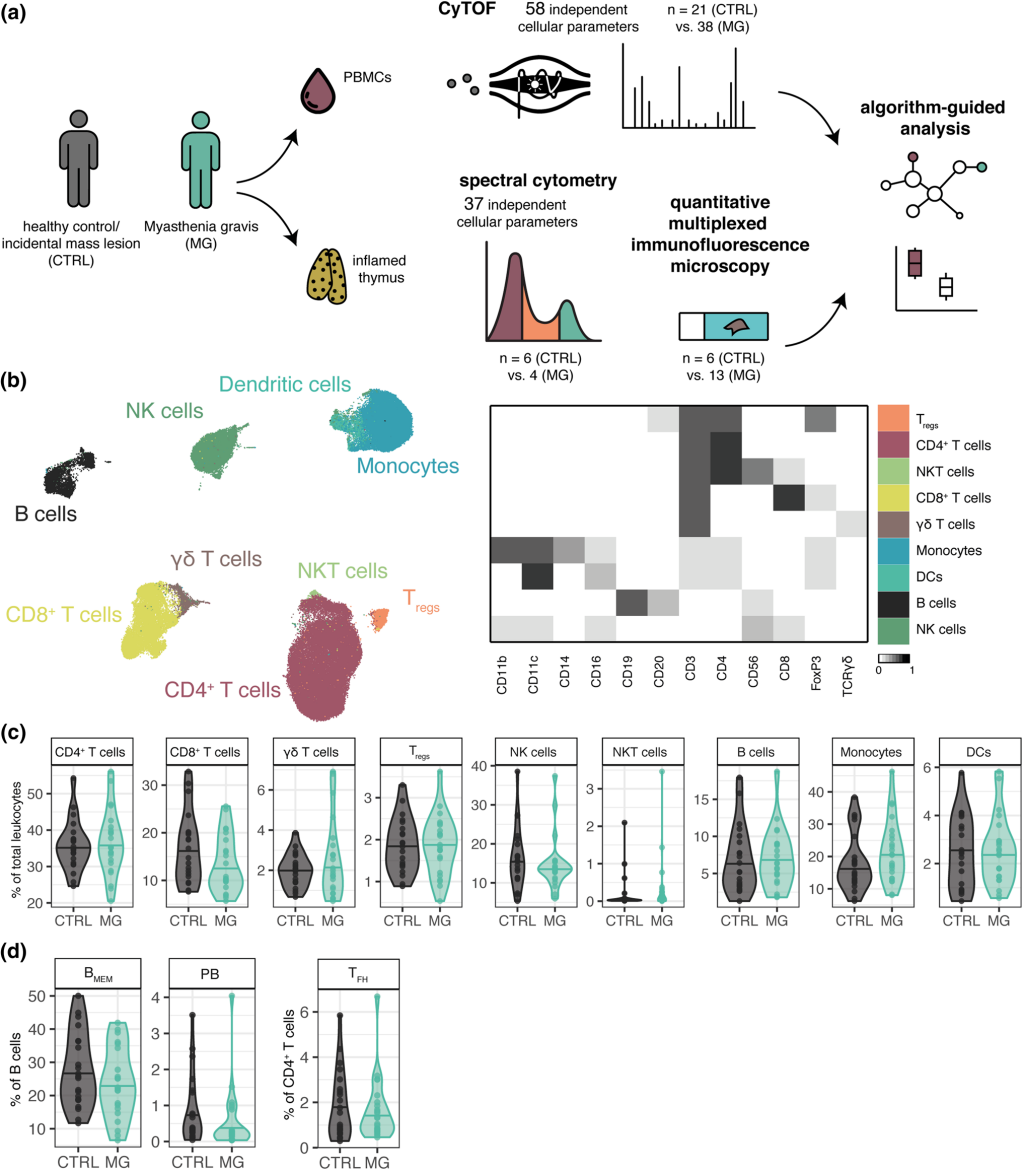
The canonical peripheral immune landscape of MG patients does not differ from that of healthy individuals. **a** Cryopreserved peripheral blood mononuclear cells (PBMCs) from myasthenia gravis patients (MG, *n* = 38) and healthy controls (CTRL, *n* = 21) were labeled with a panel of antibodies recognizing either surface markers or intracellular cytokines (following brief antigen-independent restimulation) and data acquired by CyTOF. Thymic leukocytes from MG patients (*n* = 4) and non-MG incidental mass lesion controls (*n* = 6) were analyzed in a similar manner by partially overlapping spectral flow cytometry panels. Thymic tissue sections of MG patients (*n* = 13) and non-MG controls (*n* = 6) were analyzed by quantitative multiplexed immunofluorescence microscopy. The resulting datasets were analyzed using a data-driven high-dimensional approach using supervised and unsupervised machine-learning algorithms. **b** UMAP of 100,0000 cells randomly sampled from the combined dataset. Color code indicates FlowSOM clustering and manual annotation according to lineage marker expression profiles presented in the heatmap. *T*_*regs*_ regulatory T cells, *DCs* dendritic cells, *NK* natural killer. **c** Violin plots showing the frequency of the FlowSOM-generated immune clusters in healthy controls and MG patients that did not receive immunomodulatory treatment. **d** Violin plots showing the frequency of memory B cells (B_MEM_), plasmablasts (PB) and peripheral follicular T helper cells (T_FH_) in healthy controls and MG patients obtained by subclustering the B cell and CD4^+^ T cell compartments. Violin plots contain a bold horizontal line depicting the respective group mean. If not indicated, the differences between experimental groups were statistically not significant (*p* > 0.05) using a nonparametric Mann–Whitney–Wilcoxon test with a false-discovery correction according to the Benjamini–Hochberg approach

To generate a comprehensive immune landscape of the systemic immune compartment in MG, we first analyzed peripheral blood mononuclear cell (PBMC) samples from newly diagnosed steroid-free MG patients and compared them to age- and sex-matched healthy controls (Fig. 1a, Supplementary Data 1). Peripheral blood leukocytes were barcoded and interrogated by partially overlapping mass cytometry panels to fine-map immune populations with regards to their lineage, trafficking and activation marker expression and the production of 13 different cytokines at single-cell resolution (Fig. 1a, Supplementary Fig. 1a). We then randomly sampled a portion of cells from the combined dataset (100,000 cells) and projected them onto a Uniform Manifold Approximation and Projection (UMAP) [34] for dimensionality reduction (Fig. 1b). FlowSOM, an unsupervised clustering method [19], was employed to describe the most abundant immune populations in the blood: CD4^+^ T cells, CD8^+^ T cells, γδ T cells, T_reg_ cells, natural killer (NK) cells, NKT cells, B cells, monocytes and dendritic cells (Fig. 1b). When we compared the frequencies of these immune cell populations, we found that they were comparable between age- and sex-matched healthy controls and MG patients (Fig. 1c, Supplementary Fig. 1b). Even when we conducted a subset analysis, including those populations previously associated with autoantibody production, such as plasmablasts, memory B cells and T_FH_ cells (Supplementary Fig. 1c), we again did not reveal any frequency differences between patient and control groups (Fig. 1d). Thus, the canonical immune cell composition of blood from MG patients closely resembles that of healthy individuals; this suggests that the pathogenic changes in MG are underpinned either through a functional disturbance of systemic immunity, or through alterations to a locally restricted immune compartment such as the thymus.

### Peripheral Th cells of MG patients are characterized by reduced cytokine polarization

**Fig. 2:**
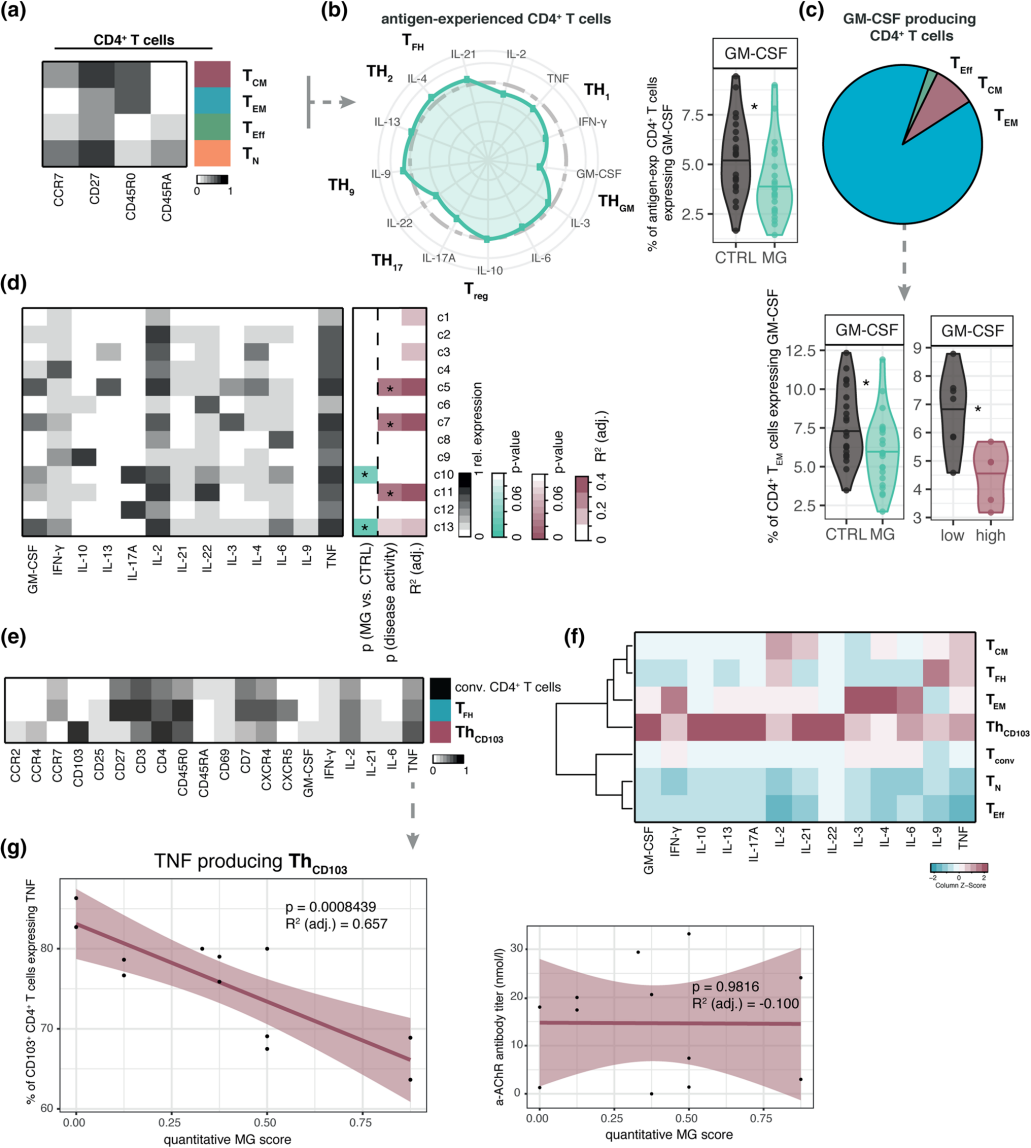
Reduced cytokine polarization and systemic TNF-producing CD103^+^ T cells represent an MG-specific signature. **a** Heat map showing relative expression of the indicated activation markers within naïve, memory and effector subsets of CD4^+^ T cells obtained by FlowSOM clustering. **b** Radar plot (left panel) representing the cytokine profile of antigen-experienced CD4^+^ T cell subsets (T_Eff_, T_EM_ and T_CM_). The colored line indicates the Cohen’s *d* effect size for each cytokine (MG vs. CTRL) as a deviation from the gray dashed reference line. Th cytokine profiles were manually annotated based on partially overlapping key cytokines. The violin plot (right panel) shows the frequency of GM-CSF^+^ cells among antigen-experienced CD4^+^ T cells in control and MG patients. *T*_*CM*_ central memory T, *T*_*EM*_ effector memory T, *T*_*Eff*_ effector T, *T*_*N*_ naive T. **c** Subset composition of the GM-CSF-producing Th cell population within all patients’ blood (MG and CTRL; upper panel); and relative abundance of GM-CSF-producing cells within the CD4^+^ T_EM_ population (bottom panel) in control and MG patients (left), and in low and high disease severity newly diagnosed treatment-naïve MG patients (right). Clinical disease severity was determined by the modified quantitative MG score, with low disease severity scoring < 0.5, and high disease severity ≥ 0.5. **d** Cytokine expression analysis of CD4^+^ T_EM_ cells. FlowSOM yielded 13 cytokine expressing clusters (c1–c13; determined by consensus clustering). Corresponding expression profiles (left box) as well as statistical parameters (right box) are displayed. Blue color indicates high significance (low *p* value) for the comparison of treatment-naïve MG patients vs. CTRL, red color indicates high significance and high *R*^2^ value, respectively, for the correlation with the continuous clinical disease severity. **e** Heatmap comparing follicular Th cells (T_FH_) and CD103^+^ Th cells (Th_CD103_) to conventional Th cells. **f** Row-normalized heatmap of cytokine positivity for 13 detected cytokines among peripheral Th subsets for all patients present in the cohort. **g** Correlation between the frequency of TNF-producing Th_CD103_ cells (left panel) or serum anti-AChR antibody titer (right panel) and the modified quantitative MG score as a measure of clinical disease severity for newly diagnosed patients neither receiving immunomodulatory nor symptomatic treatment. Violin plots contain a bold horizontal line depicting the respective group mean. If not indicated, differences between experimental groups were statistically not significant (*p* > 0.05) using a nonparametric Mann–Whitney–Wilcoxon test with a false-discovery correction according to the Benjamini–Hochberg approach. *p < 0.05. For correlation analysis, statistical parameters were obtained using a linear regression model. Shaded areas in **g** represent the 95% confidence interval

We then focused on functional immune profiling by identifying the T cell subsets present in MG and healthy blood, and detecting the cytokines produced by them. We found that CD4^+^ T cell effector subsets (Fig. 2a, Supplementary Fig. 2a) existed at comparable frequencies in MG patients and healthy controls (Supplementary Fig. 2b). However, when we compared cytokine profiles of antigen-experienced Th subsets [effector Th (T_Eff_), effector memory Th (T_EM_) and central memory Th (T_CM_)] we found that MG patients had significantly fewer granulocyte–monocyte colony-stimulating factor (GM-CSF)-expressing cells than healthy controls (Fig. 2b, Supplementary Fig. 2c). The vast majority of GM-CSF-producing Th cells were from the T_EM_ population (Fig. 2c). Strikingly, although the frequency of GM-CSF-producing CD4^+^ T_EM_ cells was decreased in MG patients in general, it was especially low in the blood of newly diagnosed treatment-naïve MG patients with highly active disease compared to those with mild disease, where MG severity is assessed by the modified quantitative MG score [6] (Fig. 2c, Supplementary Fig. 2d). Thus, it appeared that the GM-CSF-producing CD4^+^ T_EM_ frequency is significantly dysregulated in MG.

To explore this possibility, we used CD4^+^ T_EM_ cytokine expression data to visualize clusters of cells with specific patterns of cytokine production in the blood of treatment-naïve MG patients and healthy controls. We identified 13 clusters of cytokine-producing cells, of which three were significantly associated with disease severity and two with the disease per se (Fig. 2d, Supplementary Fig. 2e). Interestingly, all five differentially regulated T_EM_ clusters were those that produced GM-CSF, in combination with tumor necrosis factor (TNF) and interleukin-2 (IL-2). Taken together, despite the inflammatory nature of autoimmune MG, these data clearly show that a general GM-CSF contraction within the T_EM_ population, rather than a specific cytokine profile, characterizes the blood of MG patients.

### Systemic TNF-producing CD103^+^ Th cells represent a disease-specific signature population, which negatively correlates with the clinical disease severity

Alongside dysregulation of GM-CSF-producing T_EM_ cells, we analyzed trafficking molecules in the Th compartment and found two phenotypically distinct clusters of memory Th cells that were characterized by exclusive expression of the trafficking molecules CXCR5 (T_FH_) or CD103 (Th_CD103_) (Fig. 2e, Supplementary Fig. 3a and b). We next assessed the functional properties of the identified Th cell subsets in the blood. Interestingly, the blood Th_CD103_ population, which has recently been described as a subset of formerly tissue-resident Th cells that have reentered the circulation [29], demonstrated higher frequencies of GM-CSF-, TNF-, IL-17A-, IL-22-, IL-21-, IL-13- and IL-10-producing cells than any other subset (Fig. 2f, Supplementary Fig. 3c). Moreover, principal component analysis of the cytokine profiles of the Th subsets demonstrated a remarkable heterogeneity in the Th_CD103_ compartment, which partially overlapped with T_FH_ cells, T_EM_ cells and T_CM_ cells (Supplementary Fig. 3d). These results indicate that this highly inflammatory subset may have versatile functional properties and a remarkable trafficking capacity, as indicated by the T_EM_ phenotype combined with expression of CCR2, CCR4, CXCR4 and CD103 (Fig. 2e).

Although, overall, MG and control blood contained similar frequencies of Th_CD103_ and T_FH_ cells across all Th cell populations (Supplementary Fig. 3e), when we compared MG patients with low and high disease burden, we uncovered a significant inverse correlation between the frequencies of TNF-producing Th_CD103_ cells (but for none of the other 12 cytokines analyzed; Supplementary Fig. 4a) and clinical disease severity (Fig. 2g, Supplementary Fig. 4b). In contrast, anti-AChR autoantibody titer in serum, which is the routine blood biomarker for the diagnosis of MG, did not correlate with disease severity (Fig. 2g, right panel). Lastly, we ruled out the possibility that GM-CSF-producing Th_CD103_ cells, which demonstrate an overlap with GM-CSF-expressing T_EM_ cells, are solely responsible for the contraction of GM-CSF-expressing Th cells observed in MG (Supplementary Fig. 4c–f).

Collectively, while the overall immune composition in the blood of MG patients did not differ from healthy individuals, we observed consistently lower frequencies of certain patrolling T_EM_ subsets in MG patients, such as GM-CSF-expressing Th cells (Th_GM_) and Th_CD103_ cells. This contraction of an inflammatory signature was particularly pronounced in patients with a high disease burden, again counter to our current understanding of the inflammatory processes in MG.

### Thymi of MG patients are heavily infiltrated by Th cells and B cells

**Fig. 3:**
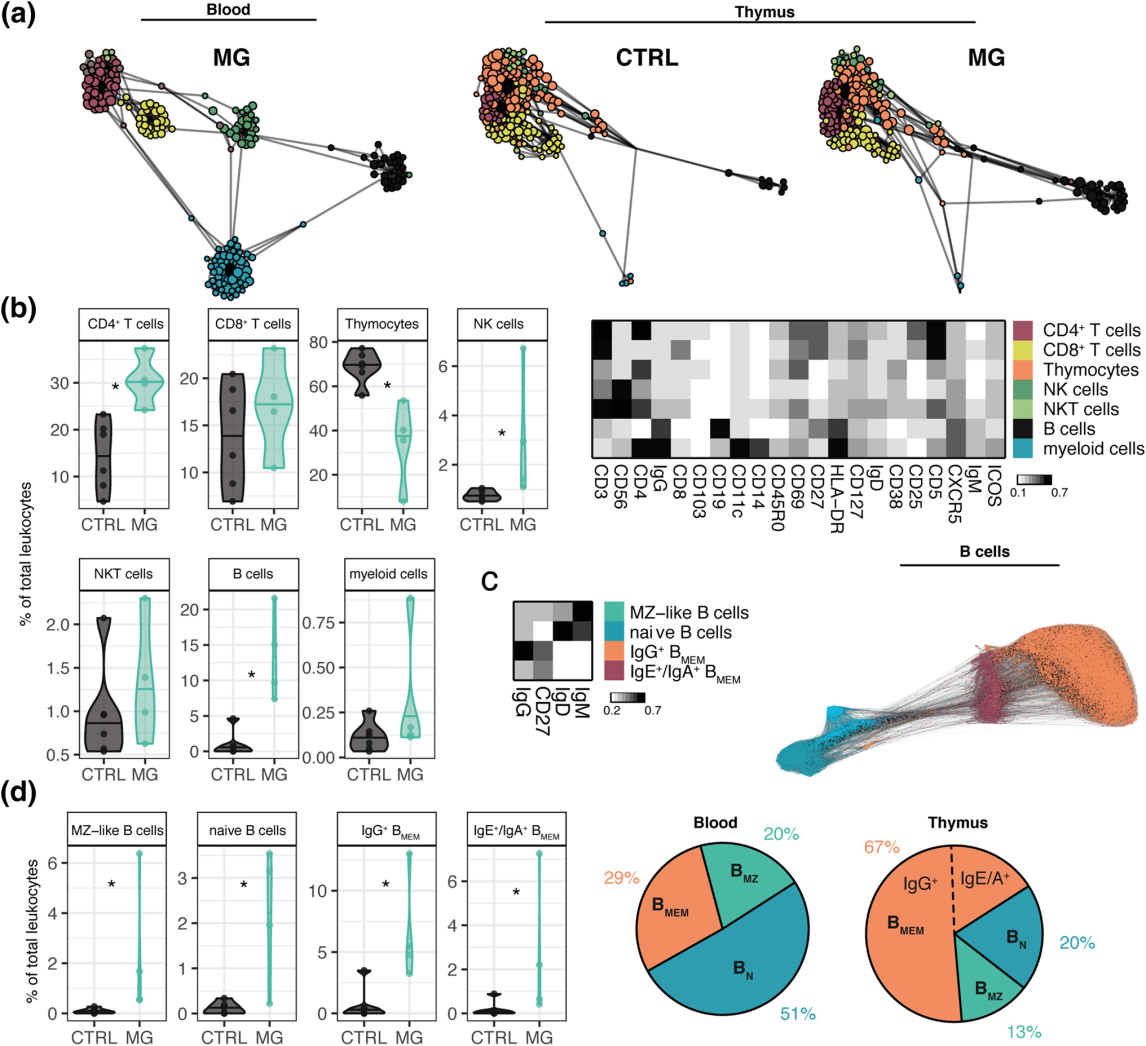
Thymi of MG patients are infiltrated by Th cells and B cells. **a** Scaffold of the mass cytometry run of the peripheral immune compartment of MG patients and the corresponding maps of the thymic leukocyte landscape of MG patients and non-MG controls determined by flow cytometry. Heatmap below depicts FlowSOM clustering of thymus samples. **b** Violin plots showing frequency of FlowSOM-generated thymic immune clusters for MG patients and incidental non-MG mass lesions. **c** Force-directed layout depicting the network of B cells present in the thymus. Color coding indicates FlowSOM clustering into subpopulations as shown in the heatmap (right panel). *MZ* marginal zone, *B*_*MEM*_ memory B. **d** Violin plots showing frequencies of different B cell subpopulations in the thymus of MG patients and non-MG controls. Pie charts depict the median frequency of peripheral and thymic B cell populations in MG patients. Violin plots contain a bold horizontal line depicting the respective group mean. If not indicated, differences between experimental groups were statistically not significant (*p* > 0.05) using a nonparametric Mann–Whitney–Wilcoxon test with a false-discovery correction according to the Benjamini–Hochberg approach. *p < 0.05

Given the specific contraction of circulatory Th_CD103_ and Th_GM_ cells in patients with high disease severity, we speculated whether these cells accumulate in the inflamed thymi of MG patients, characteristic for MG pathology. The thymus of MG patients harbors ectopic germinal centers and high frequencies of autoreactive T and B lymphocytes [4, 5, 41]. To test the notion that the thymus represents the niche wherein those cells reside, we collected thymic tissue from MG patients and sex- and age-matched controls with incidental thymic mass and similar thymus pathology, but without MG (Supplementary Data 2). We compared the CyTOF analysis of the PBMC compartment with the spectral flow cytometry data of the thymic immune landscape by applying the Scaffold framework technique [39]. This computational tool used the immune landscape of the blood as a reference network onto which the thymic leukocytes were projected (Fig. 3a). FlowSOM clustering was employed in a similar fashion as for the blood analysis to obtain comparable cell populations. Apart from mature leukocytes (CD4^+^ and CD8^+^ T cells, B cells, NK cells, NKT cells and myeloid cells), we identified a cluster of developing thymocytes in thymi (Fig. 3a). Notably, and in line with previous reports supporting a key role for the thymus in maintaining autoreactivity against AChR during MG, we observed that compared to control tissue, MG thymi had relatively higher frequencies of B cells and Th cells, but not cytotoxic CD8^+^ T cells (Fig. 3b). Furthermore, we observed a significant increase in NK cell frequency in MG thymi (Fig. 3b). Control thymi were matched for age (see Supplementary Data 2), thus, the relative decrease of immature thymocytes in thymi of MG patients was likely caused by an influx of circulating leukocytes. First, focusing on B cells (Fig. 3c, d), we observed a significant increase in all subpopulations, including class-switched IgG^+^ and IgE^+^/IgA^+^ memory B cells, in the thymi of MG patients compared to controls (Fig. 3d). Compared to the peripheral blood of MG patients, the MG thymi contained relatively more memory B cells, supporting the concept of a well-defined local inflammatory process underlying autoimmunity in MG (Fig. 3d, right panel).

### Medullary regions of the thymi of MG patients are infiltrated by Th_CD103_ and Th_GM_ cells

**Fig. 4:**
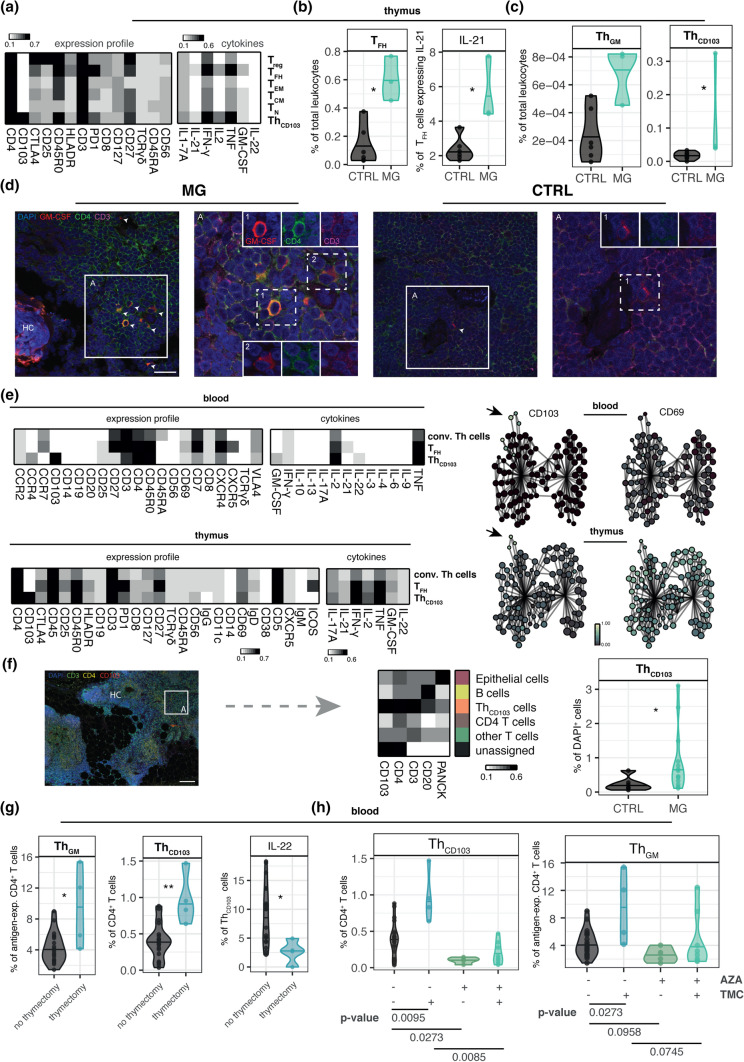
Signature Th cells are enriched in medullary regions of MG thymi and rebound in the blood after thymectomy. **a** Heatmap of surface markers and cytokine expression profiles of FlowSOM-generated Th subsets. **b** Violin plots comparing the frequencies of T_FH_ cells and the corresponding frequency of IL-21 expression among T_FH_ cells in MG patients vs non-MG patients. **c** Violin plot showing the frequency of GM-CSF expressing Th cells (Th_GM_) and Th_CD103_ cells in the thymi of MG and non-MG patients. **d** Immunofluorescence labeling of medullary thymic regions showing GM-CSF (red), CD4 (green), CD3 (magenta) and DAPI (blue). Samples from 7 MG patients and 5 non-MG controls were analyzed. Scale bar: 30 µm. Images of the single labels are enlargements of specified regions. Representative images of two independent experiments (1 slide with 3 sections/patient each) are shown. Left: MG, early onset, female MG patient without immunosuppressive therapy and thymus follicular hyperplasia, right: CTRL, patient with incidental mass, residual thymus tissue. *HC* Hassall’s corpuscle. **e** Heatmap of surface markers and cytokine expression profiles of FlowSOM-generated Th subsets in blood and thymus (left panel). Thymic expression profiles from both panels were clustered analogously using FlowSOM and displayed as combined expression profiles. Scaffold of the blood and thymic Th cell compartment in MG patients (right panel). Color overlay depicts expression of CD103 and CD69. **f** Heatmap of expression profiles of FlowSOM-generated cell clusters for quantitative immunofluorescence data of thymic regions after cell segmentation (middle panel). Colors correspond to the populations shown in Fig. S5f. Violin plots showing the frequency of Th_CD103_ cells within DAPI^+^ cells of MG patients and incidental mass lesion controls (right panel). *PANCK* pan-Cytokeratin. **g** Violin plots showing the frequency of Th_GM_ and Th_CD103_ cells, and the frequency of IL-22 production within Th_CD103_ cells, in the blood of MG patients with or without thymectomy that did not receive further immunomodulatory treatment. **h** Violin plots showing the frequency of Th_CD103_ and Th_GM_ cells in the blood of MG patients without treatment or with thymectomy (TMC) and/or azathioprine (AZA) treatment. Violin plots contain a bold horizontal line depicting the respective group mean. If not indicated, differences between experimental groups were statistically not significant (*p* > 0.05) using a nonparametric Mann–Whitney–Wilcoxon test with a false-discovery correction according to the Benjamini–Hochberg approach. *p < 0.05; **p < 0.01

In accordance with the marked infiltration of class-switched memory B cells, we found increased frequencies of total and IL-21-expressing T_FH_ cells in MG patients compared to controls (Fig. 4a, b, Supplementary Fig. 5a). Enrichment of thymic T_FH_ cells has been described by other studies before and supports the notion of local B cell-supporting T cell pathology [11, 38]. When we measured the overall in situ cytokine profile of antigen-experienced Th cells, we did not find significant alterations in the inflamed thymus of MG patients compared to control thymi (Supplementary Fig. 5b); however, we found a strong trend (*p* = 0.052) towards increased frequencies of Th_GM_ cells, the cell population that was specifically contracted in the systemic immune compartment of MG (Fig. 4c). Moreover, we could find Th_GM_ cells in medullary regions of hyperplastic thymi of MG patients (Fig. 4d). Focusing on the other Th cell subset that appeared dysregulated in the blood, we observed that Th_CD103_ signature cells were specifically enriched in the MG thymus. To follow the notion that thymic Th_CD103_ cells represent highly inflammatory tissue-resident memory Th cells that could reenter the circulation and give rise to Th_CD103_ cells found in the peripheral blood, we performed a detailed phenotypic comparison between thymic and blood Th_CD103_ cells, T_FH_ cells and conventional Th cells. In line with previous reports [30, 45, 48], thymic Th_CD103_ cells expressed the tissue-retention molecule CD69 that was absent in the blood counterpart, but demonstrated a shared activated memory phenotype with higher expression of IL-2 and TNF than T_FH_ or conventional Th cells (Fig. 4e, Supplementary Fig. 5c). Furthermore, expression of CCR4 in the Th_CD103_ cluster in the blood (Fig. 4e) supported the migratory capacity of these cells and their ability to sense CCL17 and CCL22; these chemokines are abundant in the thymic medulla where they are secreted by macrophages and dendritic cells and provide medullary entry sites for thymocytes during thymic development [7, 24, 25]. Interestingly, thymic Th_CD103_ cells shared characteristics with T_FH_ cells such as high PD-1 expression and secretion of IL-21 but could be distinguished from T_FH_ cells due to the absence of ICOS, the higher expression of IL-17A and IL-2 and the exclusive expression of CD103 (Fig. 4e). To increase the number of analyzed samples, we validated the enrichment of Th_CD103_ cells in thymic sections of 13 MG patients and 6 controls by multiplexed quantitative histology and located Th_CD103_ cells within B cell-rich medullary regions of MG thymi (Fig. 4f, Supplementary Fig. 5d–f, Supplementary Data 3). Together with the contraction of TNF-producing CD103^+^ Th cells in the systemic compartment of MG patients, these findings support a pathogenic role of tissue-resident Th cells in locally driven B cell pathology in MG.

### Th_CD103_ and Th_GM_ cells rebound in the blood of MG patients after thymectomy

The specific contraction of Th_CD103_ and Th_GM_ cells in the systemic immune compartment in conjunction with an expansion of the signature cells in the inflamed thymus of MG patients points towards a pathogenic local retention of those cells within the inflamed thymus, rather than a global dysregulation of cell frequencies per se. To test this hypothesis, we measured the frequencies of the identified signature subsets in the blood after therapeutic thymectomy; the surgical removal of the inflammatory thymic niche. In thymectomized MG patients, we observed significant decreases in the relative frequency of T_Eff_ coupled with expansion of T_EM_ populations in blood compared to no thymectomy control MG patients (Supplementary Fig. 6a). Notably, the increase of T_EM_ cells after thymectomy followed the opposite trend when comparing MG patients to healthy controls (Supplementary Fig. 2b). Most importantly, we found systemic expansion of the inflammatory signature Th_CD103_ and Th_GM_ populations in athymic patients (Fig. 4g), even though timepoints for blood sampling after thymectomy ranged from several months to several years (Supplementary Data 1). Interestingly, the high levels of the thymus tissue regenerative cytokine IL-22 [14] produced by Th_CD103_ cells were markedly reduced in MG patients previously treated with thymectomy (Fig. 4g, Supplementary Fig. 6b). Since thymectomy released signature cells from the thymus, which could spread to secondary niches such as lymph nodes and bone marrow via blood circulation [16, 17], we lastly investigated whether Th_CD103_ and Th_GM_ cells in the blood were suppressed by standard MG immunotherapy. Due to the lack of targeted immunotherapies in MG, broad immunosuppressive agents like azathioprine, a purine synthesis inhibitor, are administered to stabilize the patients [20]. Coinciding with immunosuppression, we observed a significant reduction in the relative frequency of Th_CD103_ cells in the blood in patients treated with azathioprine (Fig. 4h). Azathioprine even suppressed the rebound of Th_GM_ and Th_CD103_ cells driven by thymectomy (Fig. 4h). However, the biggest effect of azathioprine treatment was on the NK cell compartment, especially reducing the CD56^dim^ NK cell population (Supplementary Fig. 6c and d). This observation is in line with the increased risk of azathioprine-treated patients to develop recurrent viral infections and virus-induced malignancies [31] highlighting the need for more tailored therapeutic interventions.

## Discussion

Our single-cell profiling study provides novel insights into Th cell-driven MG immunopathology in the thymic niche, and its reflection in the systemic circulation. Using unsupervised machine-learning tools, we identified two subsets of Th cells: Th_GM_ and Th_CD103_, which were enriched in the inflamed thymi of MG patients, and thus, appeared in lower numbers in the blood. Moreover, the frequencies of both populations correlated with the clinical disease severity of MG patients, unlike the amount of serum anti-AChR antibodies [37]. In line with the inflammatory phenotype of Th_GM_ cells, a previous study reported GM-CSF as a hallmark cytokine expressed in AChR-reactive Th cells in MG patients [10]. The previous study incorporated antigen-specific T cell libraries and found that, alongside GM-CSF, autoreactive T cells also produced IL-17 and IFN-γ. In addition, we pinpointed IL-22 expression to our signature Th_CD103_ cells, similar to previous findings in skin Th_CD103_ cells [29]. Of note, IL-22 receptor expression is essentially restricted to epithelial and stromal cells, including those of the thymic tissue [13, 14]. Some data suggest that IL-22 has a dual role locally, as it is linked to chronic inflammation but is also essential for thymic regenerative processes limiting tissue injury [14, 46].

Surgical removal of the thymus has long been a standard treatment for MG, and the recently published multicenter randomized MGTX phase 3 trial confirmed the beneficial long-term effects of this procedure compared to oral steroids alone in MG patients [42]. It is thought that its efficacy is based on the removal of ectopic germinal centers and local pathogenic cells that drive the production of anti-AChR autoantibodies [20]. In our study, the signature populations rebounded in the blood after surgical removal of the inflammatory niche supporting the notion of pathological inflammatory leukocyte retention in the thymus during MG. Robat-Jazi and colleagues found significantly decreased IL-22^+^ Th cells in the blood of MG patients after thymectomy (compared to before thymectomy) [35], which is in accordance with our results of reduced IL-22 production by Th_CD103_ cells after removal of the thymus.

From a clinical viewpoint, our study identifies the TNF-producing Th_CD103_ cell subset as a promising candidate as a cellular marker of disease severity in MG which should be validated in further studies. CD103 (integrin αEβ7) normally retains T cells within epithelial-rich tissues via binding to E-cadherin which is highly expressed in thymic medullary regions [32]. Several investigations have shown that the presence of such cytokine-secreting CD103^+^ tissue-resident memory Th cells is associated with poor outcomes in chronic inflammatory skin diseases such as psoriasis [8], vitiligo [12] and alopecia areata [43]. In addition, in the gut, a potential pro-inflammatory role has been allocated to the Th_CD103_ subset [33]. More recently, Zundler and colleagues reported that CD103^+^ tissue-resident memory Th cells accumulated in the mucosa of patients with inflammatory bowel disease, and that the presence of CD69^+^ Th_CD103_ cells was predictive of flares [48].

Even though the expression of CD103 on T cells has been clearly associated with tissue residency, the function of the circulating CD103^+^ Th population remains considerably less defined. A recent study shed more light on their enigmatic role, demonstrating that skin-resident CD69^+^ CD103^+^ Th cells can downregulate the tissue-retention marker CD69, reenter the circulation and migrate to secondary human skin locations [29]. This is in accordance with the predominant T_EM_ phenotype, versatile cytokine profile, the broad expression of trafficking receptors and the exclusive expression of CD69 in the tissue but not in the circulation that we observed in the Th_CD103_ cell subset. One might speculate that the circulating properties of both Th subsets represent a shuttle between the origin of disease pathology and secondary sites of chronic pathogenic antibody production (in particular after thymectomy) as suggested by murine xenograft and local infection models [28, 29]. As a proof of principle, we demonstrated that both dysregulated memory Th subsets, Th_GM_ and Th_CD103_, were indeed effective targets of long-term immunosuppression by azathioprine in steroid-free MG patients. Counter-intuitively, both MG treatment approaches, thymectomy and azathioprine, modulated the frequency of signature cells in the blood in opposite directions, indicating two distinct mechanisms of action. Thymectomy abrogates the retention of inflammatory cells in the thymus thereby increasing signature Th cells in the blood, whereas azathioprine halts cell replication and triggers apoptosis in leukocytes [9]: the combination of these therapies, therefore, both removes pathogenic cells in the thymus and decreases Th signature cells in the circulation. Of note, perioperative high-dose immunosuppression has been shown by others to give a favorable clinical response compared to thymectomy alone [44].

Therefore, novel therapeutic interventions aimed at depleting or reducing Th_GM_ or Th_CD103_ may improve the outcome of MG treatment. One such approach that is currently being developed is the use of specific anti-integrins, controlling both inflammatory effects and immune cell trafficking: Etrolizumab, a monoclonal antibody targeting the two integrins CD103 and α4β7, led to clinical remission in patients with ulcerative colitis in a phase 2 trial, and consecutive phase 3 trials are ongoing [40].

Additional work is required to validate the applicability of the identified signature as a therapeutic target or biomarker for MG disease progression and response to therapy. Due to the heterogenous nature of the disease, it remains unclear whether the identified Th cell signature is restricted to anti-AChR-antibody-positive MG patients, that we exclusively focused on in this study, or if it can be generalized to other MG forms, not involving the thymus. For example, anti-Muscle-Specific Kinase (MuSK)-positive antibody and seronegative MG patients were not included in this study. In line with this notion, a subject for future studies is to further validate the specificity of the signature compared to other autoimmune diseases such as multiple sclerosis, neuromyelitis optica spectrum disorders or systemic lupus erythematosus. Even though a direct comparison between the disease signatures is pending, previous studies regarding disease signatures across autoimmune diseases suggest that there is no major overlap [18, 22].

Despite these major limitations, our report represents, to our knowledge, to date the most comprehensive map of the systemic immune compartment and the disease-driving thymus in MG patients. Moreover, it represents a major conceptual advance with the potential to be translated into the clinic, upon further validation using large-scale studies, for the therapeutic targeting of MG, as well as biomarker development.

## Materials and methods

### Healthy donors and MG patient samples

The study was approved by the Ethics Commission Zurich, Switzerland. MG patients (*n* = 21 healthy controls and *n* = 38 MG patients) were recruited and upon written informed consent blood and thymus samples were obtained at the Neuromuscular Center and the Department of Thoracic Surgery of the University Hospital Zurich, Switzerland. PBMC and serum samples were isolated at the University of Zurich within 12 h of blood collection. Fresh thymus tissue was obtained from patients undergoing elective thymectomy, in which thymus is routinely discarded (*n* = 6 non-MG controls versus *n* = 5 MG patients). Furthermore, we retrospectively examined formalin-fixed thymectomy samples provided by the tissue biobank of the Department of Pathology (University Hospital Zurich) by multiplexed immunofluorescence (Supplementary Data 3; *n* = 6 non-MG controls and *n* = 13 MG patients). Standard histopathological analysis of thymus tissue was performed by a clinical pathologist independent of the study. Samples from both sexes were included in the study. Ages ranged from 23 to 91 years. Diagnosis of MG was based on typical clinical symptoms, a positive anti-AChR antibody test in the serum, a positive electrophysiological measurement, and response to treatment with acetylcholinesterase inhibitors. The diagnosis was confirmed by a neurologist with experience in the care of these patients. No seronegative or anti-MuSK-positive MG patients were included in the study. Healthy controls were age- and sex-matched, had no evidence of acute or chronic infection and were not receiving immunomodulatory therapy. In addition, patients with an (incidental) thymic mass or thymoma and without MG were included in the study (Supplementary Data 2). For the blood comparison of MG patients to healthy controls, only patients that were not treated with immunomodulatory drugs or thymectomy were considered (*n* = 21 healthy controls and *n* = 22 MG patients). For correlations with the patients’ clinical disease, severity exclusively untreated MG patients (excluding symptomatically treated patients; *n* = 12) were considered and the continuous relative quantitative Besinger score was used as a measure of disease severity [6]. The Besinger score is a quantitative MG scoring system that has been adapted and slightly expanded by the MG Foundation of America (MGFA) Task Force for therapy studies (QMG score) [27] and contains similar sub scores (except hand-held dynamometer measurements). When using disease severity as a categorical value, patients demonstrating a relative Besinger score ≥ 0.5 were considered as having high disease severity. For the comparison of thymectomy and/or azathioprine-treated/untreated MG patients, only patients that did not receive further immunomodulatory treatment were considered for the analysis (*n* = 20 non-thymectomized versus *n* = 4 thymectomized; *n* = 27 azathioprine-untreated versus *n* = 12 azathioprine-treated).

### Serum anti-AChR autoantibody level measurement

Serum patient anti-AChR autoantibodies were measured at the Department of Clinical Immunology (University Hospital, Zurich) using an ^125^I radioimmunoassay based on reactivity against fetal and adult nicotinic AChR (DLD Diagnostika), as part of the routine diagnostic procedure.

### Leukocyte isolation from blood and thymic tissue

Blood samples were diluted in PBS and PBMC fraction was isolated using SepMate 50 tubes (Stemcell Technologies) and human Lympholyte Separation Medium (Cedarlane). To ensure comparability with PBMC samples, a similar protocol for thymic leukocyte isolation was chosen. In brief, thymic tissue was placed on ice immediately after surgery and processed. A single-cell suspension was achieved by cutting the tissue using a scalpel and syringes. The cell suspension was washed and the leukocyte fraction isolated by density gradient centrifugation using human Lympholyte Separation Medium (Cedarlane). The resulting lymphocyte fraction was washed, cryopreserved in 10% DMSO in fetal calf serum (FCS; Biochrom) and stored in the vapor phase of a liquid nitrogen tank until further analysis.

### Ex vivo reactivation of PBMCs

Short-term reactivation of cryopreserved PBMCs or thymic leukocytes and subsequent cytometry analysis were performed as described previously [22]. In short, leukocytes were stored in liquid nitrogen and thawed in a 37 °C water bath before use. Cells were resuspended in cell culture medium [RPMI-1640, 10% FCS (Biochrom), and 1 × l-glutamine and 1 × penicillin/streptomycin (both Life Technologies)] supplemented with 1:10,000 benzonase (Sigma–Aldrich), centrifuged (300×*g*, 7 min; 24 °C) and washed twice with cell culture medium. Samples then underwent antibody labeling, or in the case of intracellular cytokine detection, were rested overnight at 37 °C and restimulated with 50 ng ml^−1^ phorbol 12-myristate 13-acetate (Sigma–Aldrich) and 500 ng ml^−1^ ionomycin (Sigma–Aldrich) in the presence of 1 × Brefeldin A and 1 × Monensin (both BD Biosciences) for 4 h at 37 °C before surface marker antibody labeling, fixation, permeabilization and intracellular cytokine antibody labeling.

### Antibodies

For mass cytometry, monoclonal anti-human antibodies (Supplementary Table 1) were purchased either conjugated to heavy-metal isotopes (Fluidigm) or were conjugated in house using the MaxPar X8 chelating polymer kit (Fluidigm). Flow cytometry antibodies were purchased already conjugated to the specified fluorochromes (Supplementary Table 2).

### Live cell barcoding for mass cytometry

To reduce inter-sample staining variability, minimize sample-handling time, and reduce instrument performance-based signal variation, we made use of a combinatorial live-cell barcoding approach using differentially conjugated anti-CD45 mAbs (Biolegend). MaxPar X8 polymers (Fluidigm) were loaded with six different palladium isotopes (102Pd, 104Pd, 105Pd, 106Pd, 108Pd, and 110Pd) and one indium isotope (115In; all from Trace Sciences International) and conjugated to anti-human CD45 mAbs (BioLegend). To exclude doublets and prevent misidentification of barcodes during debarcoding, a restricted 7-choose-3 approach was applied, resulting in 35 barcodes per mass cytometry run. Independent mass cytometry runs contained equal ratios of MG patients and healthy controls and were subject to randomization with regards to treatment, sex, and age. PBMCs were labeled with heavy metal-tagged CD45 antibodies after ex vivo reactivation at 37 °C for 25 min in cell-staining medium (CSM; RPMI-1640, 4% FCS) on an orbital shaker (500 rpm). Samples were washed twice in CSM and combined into a single reaction vessel for surface marker and cytokine detection.

### Surface and intracellular cytokine detection by mass cytometry

The barcoded sample convolute was labeled in 400 μl CSM containing the antibody mix directed against surface markers for 40 min at 37 °C on an orbital shaker (500 rpm). For dead cell discrimination, 2.5 μM cisplatin (Sigma-Aldrich) was added for 2 min on ice.

For transcription factor detection, the sample convolute was fixed and permeabilized for 40 min at 4 °C in 1X FOXP3 Fixation/Permeabilization Buffer (BioLegend). Sample was washed in permeabilization buffer [(PBS, 0.5% saponin, 2% bovine serum albumin (BSA), 0.01% sodium azide (all Sigma-Aldrich)] and nuclear staining was performed in 400 μl permeabilization buffer for 1 h at 4 °C.

For intracellular cytokine detection the sample convolute was fixed in 1.6% paraformaldehyde (Electron Microscopy Sciences) for 1 h at 4 °C. The convolute was washed with permeabilization buffer and labeled with antibodies recognizing intracellular cytokines in 400 μl permeabilization buffer for 1 h at 4 °C.

In both the cases, the labeled and stained sample mix was washed and resuspended in 1X iridium intercalator solution (Fluidigm) followed by a 4 °C incubation overnight. Finally, the sample was washed twice with PBS and twice with MaxPar water (Fluidigm) before data acquisition.

### Flow cytometry sample labeling and data acquisition

Flow cytometry labeling was performed similarly as for mass cytometry. In brief, samples were labeled with 100 μl of a fluorochrome-conjugated antibody mix in PBS for 20 min at 4 °C and washed twice in PBS. For intracellular cytokine detection, samples were fixed for 20 min at 4 °C using 100 μl Cytofix/Cytoperm (BD Biosciences). Cytokine staining was performed in 100 µl antibody mix in permeabilization buffer at 4 °C overnight. Samples were washed twice and acquired at a Cytek Aurora Spectral Analyzer (Cytek Bioscience). Quality control on a daily basis ensured reliability and reproducibility of the machine’s performance.

Compensation matrix was corrected in FlowJo (TreeStar) and samples were gated into live CD45^+^ singlets and exported to the R analysis framework.

### Mass cytometry acquisition and data preprocessing

Barcoded and labeled sample data were acquired on a CyTOF 2.1 mass cytometer (Fluidigm). Instrument quality control and tuning was performed on a daily basis. Acquisitions from two independent runs were normalized using five-element beads (Fluidigm) [15]. To further control for batch effects, each independent run contained one normalization control sample that was present in both runs. Live single cells in the sample convolute were identified based on event length, center, width, DNA (^191^Ir and ^193^Ir) and live/dead (^195^Pt) channels in FlowJo (TreeStar). Debarcoding was achieved by Boolean gating in FlowJo of cells exclusively bearing three barcodes. Both flow and mass cytometry data were transformed in the R environment using an inverse hyperbolic sine (arcsin) function. In case individual markers were not aligned in the normalization controls of both mass cytometry runs due to residual batch effects, cofactors of the sample convolute were adapted to achieve the same mean in staining intensity for both normalization controls. A channel-based percentile normalization using the 99.9th percentile was further applied on the transformed dataset for flow and mass cytometry data. Cytokine positivity was determined in an automated fashion by calculating the 99th percentile of the residual cytokine labeling of an unstimulated control.

### Immunohistochemistry

Thymus tissue samples were cryosectioned for immunohistochemistry using a Hyrax C60 cryostat (Zeiss). Thymus sections (10 μm) were fixed with 2% (wt/vol) paraformaldehyde (PFA) in 0.1 M phosphate buffer, pH 7.4, and acetone, washed in PBS, and blocked with PBS supplemented with 0.1% Triton X-100 and 4% normal goat serum. Subsequently, sections were incubated with the following primary antibodies (diluted in blocking solution) overnight at 4 °C: rat anti-GM-CSF antibody (BD Pharmingen, clone BVD2-21C11, 1:50), mouse anti-CD4 antibody (Biolegend, clone RPA-T4, 1:50) and rabbit anti-CD3 (NOVUS, clone SP7, 1:100). Sections were then washed in PBS and incubated with AF647-labeled goat anti-rat, AF488-labeled goat anti-mouse and AF555-labeled goat anti-rabbit secondary antibodies (Life Technologies, 1:500) overnight at 4 °C. Sections were mounted with SlowFade Gold antifade reagent with DAPI (Invitrogen). Fluorescence photomicrographs were captured with a SP5 Leica confocal laser scanning microscope (SP5; Leica) equipped with argon and helium lasers using the 40 × objective lens (oil immersion, NA1.25). Images were processed and merged by Imaris imaging software (Bitplane).

### Multiplexed immunofluorescence

Multispectral immunofluorescence was performed applying the following antibody panel to Formalin-fixed paraffin-embedded tissue sections of thymus and normal tonsil tissues: CD3 (Leica Microsystems Ltd, clone LN10, 1:50), CD4 (Leica Microsystems Ltd, clone 4B12, 1:50), CD20 (Leica Microsystems Ltd, clone L26, 1:200), CD23 (Leica Microsystems Ltd, clone 1B12, RTU), CD103 (Abcam, clone EPR4166(2), 1:500), Cytokeratin (Agilent Technologies, clone AE1/AE3, 1:100), and counterstaining with DAPI. The optimized multiplexed immunofluorescence protocols were validated against chromogenic singleplex protocols on consecutive sections of a normal human reactive tonsil tissue and thymus samples. Prior to staining, all tissue slides were deparaffinised on the Leica BOND RX automated immunostainer (Leica Microsystems) by soaking in BOND Dewax solution at 72 °C and then rehydrating in ethanol. Tyramide signal amplification-based Opal method was used in this study for immunofluorescence (IF) staining (Opal 7-Color Automation IHC Kit, Akoya Biosciences). The primary antibody conditions and order of staining determined using DAB detection were directly applied to the fluorescent assays. Unlike conventional immunohistochemistry, a chromogenic peroxidase substrate is used for antigen detection, each antibody is paired with an individual Opal fluorophore for visualization. Importantly, if biomarkers were expected to co-localize in the same cellular compartment then they were paired with spectrally separated Opals. In addition, low expressing markers were coupled to more intense Opals to facilitate spectral acquisition, and vice versa. The Opal fluorophores were used at a 1/100 to 1/200 dilutions. As such, a fluorescent singleplex was performed for each biomarker and compared to the appropriate chromogenic singleplex to assess staining performance.

All fluorescently labeled slides were scanned on the Vectra 3 at 20 × magnification using appropriate exposure times. Initially, whole slide images were scanned with all five standard epi-fluorescence filters (DAPI, FITC, Cy3, Texas Red and Cy5). Then, when MOTiF Opals were solely used, images were acquired using tile scanning with the mIF whole slide unmixing filters (DAPI + Opal 570/690, Opal 480/620/780, and Opal 520). Library slides were generated from representative tissue sections to allow for accurate unmixing of the multiplexed samples, including a slide stained for each single fluorophore, a DAPI only slide, and an autofluorescence slide wherein no antibody, Opal reagent or DAPI was applied. For quantification of tissue sections, cells were segmented based on the DAPI signal using the inForm 2.3 software (PerkinElmer) and imported into the statistical programming environment R. Downstream analysis including transformation, dimensionality reduction and cell clustering was carried out analogous to the cytometry analysis described below.

In addition, for individual representative CD103 stainings of thymi, immunohistochemistry was performed using the two-step IHC staining kit EnVision + System HRP DAKO (Glostrup, Denmark) according to the manufacturer’s instructions. Sections underwent heat-mediated antigen retrieval with Dako Target Retrieval Solution. DAB was used as the chromogen, and the sections counterstained with haematoxylin and analyzed with the light microscope Olympus BX41.

### Algorithm-guided high-dimensional analysis

The high-dimensional analysis was accomplished in the R environment. For FlowSOM clustering, 100 clusters were generated from the combined dataset and metaclustered based on the elbow point. The elbow point was determined by plotting the percentage of variance explained in relation to the number of metaclusters using the package *ConsensusClusterPlus*. UMAPs were generated using the R package *umap* with default settings [34]. Force-directed layouts were generated using the ForceAtlas2 algorithm [26] integrated in the VorteX graphical clustering environment creating unweighted edges between the nodes based on the 10 nearest neighbors [36]. Resulting graphs were further modified using the Gephi Toolkit 0.9.2. Scaffold networks were created using the improved version of the initial *Scaffold* package consisting of *grappolo*, *vite* and *panorama* [39]. Mass cytometry FlowSOM nodes of the peripheral blood were used to create the landmark nodes. All plots and visualizations were drawn using the *ggplot2* package.

### Statistical analysis

Immune cell frequencies were compared using the unpaired nonparametric Mann–Whitney–Wilcoxon test using the *stats* package. To correct for multiple testing, the Benjamini–Hochberg correction was applied [3]. Linear regression analysis was carried out using the R base function lm.

## Supplementary Information

Below is the link to the electronic supplementary material.

Supplementary file1 (XLSX 30 kb)

Supplementary file2 (PDF 29377 kb)

## Data Availability

Raw mass and spectral flow cytometry data are available at 10.17632/nkcb8nc7w8.1.

## Code availability

Code of the bioinformatic analysis pipeline is available at https://github.com/florianingelfinger/MG_publication.
