# Supplementary material for: Single-cell profiling of myasthenia gravis identifies a pathogenic T cell signature
Source: Acta Neuropathol. 2021 Mar 28;141(6):901–15. doi: 10.1007/s00401-021-02299-y (PMC8113175; doi:10.1007/s00401-021-02299-y)
Supplement: Supplementary file 2 — Supplementary file2 (PDF 29377 kb) [file 401_2021_2299_MOESM2_ESM.pdf]

Supplementary information

This file contains the following supplementary information:

Supplementary Figure 1–6

Supplementary Tables 1-2

Supplementary Data 1-3 (separate spreadsheets)

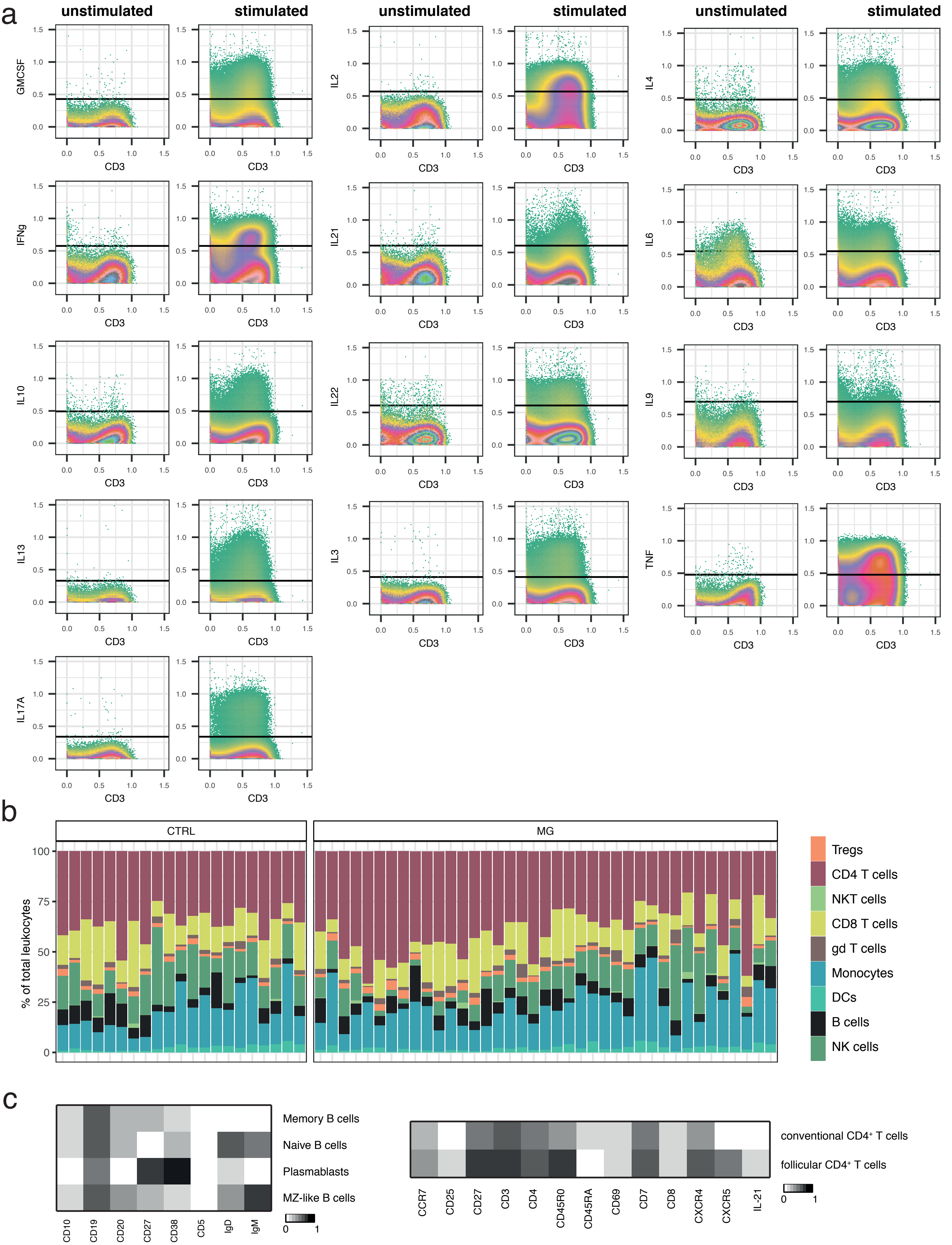
a
unstimulated
stimulated
GMCSF
IFNg
IL10
IL13
IL17A
IL2
IL21
IL22
IL3
IL4
IL6
IL9
TNF
CD3
b
CTRL
MG
% of total leukocytes
Tregs
CD4 T cells
NKT cells
CD8 T cells
gd T cells
Monocytes
DCs
B cells
NK cells
c
Memory B cells
Naive B cells
Plasmablasts
MZ-like B cells
CD10
CD19
CD20
CD27
CD38
CD5
IgD
IgM
conventional CD4+ T cells
follicular CD4+ T cells
CCR7
CD25
CD27
CD3
CD4
CD45R0
CD45RA
CD69
CD7
CD8
CXCR4
CXCR5
IL-21

Supplementary Figure 1. The circulatory major immune subsets and leukocytes associated with autoantibody production in MG patients do not differ from healthy controls.

a: Biaxial plots showing the expression of 13 different cytokines versus CD3 in blood leukocytes of the combined cohort (MG and CTRL) after ex vivo stimulation using phorbol 12-myristate 13-acetate (PMA) and ionomycin and for the unstimulated control. Horizontal line indicates gates that were set based on the 99.5th percentile of the residual cytokine staining of the unstimulated control. Gate for IL-6 was manually set based on the density of the stimulated sample.

b: Stacked bar graph showing the distribution of the most abundant leukocyte populations in the blood of each individual MG patient and healthy control enrolled in the study. Color coding refers to the indicated populations in **Fig. 1b**.

c: Heatmap showing the surface marker and intracellular cytokine expression profiles of B cell and Th cell subpopulations yielded by FlowSOM clustering as quantified in **Fig. 1d**.

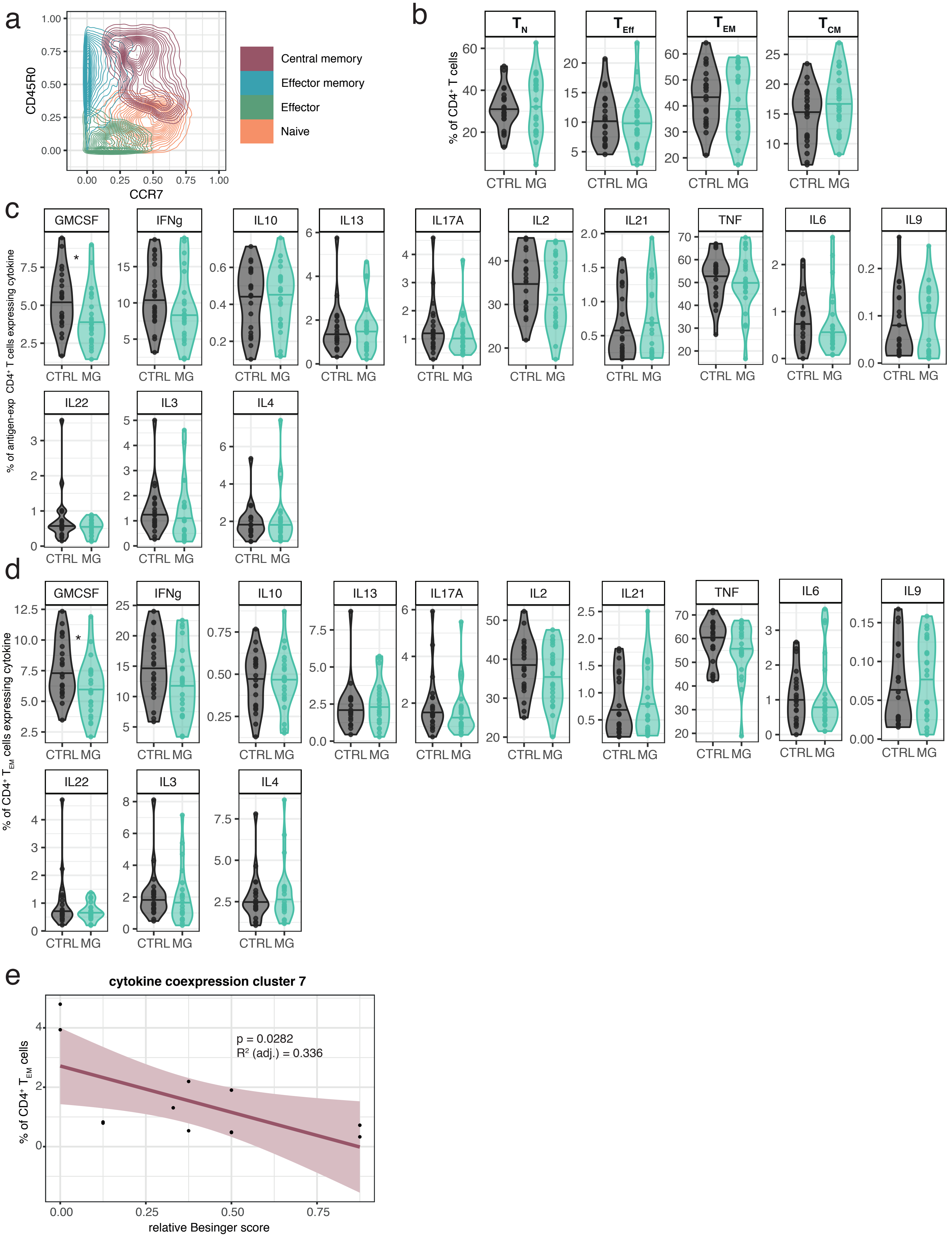

a
CD45R0
CCR7
Central memory
Effector memory
Effector
Naive
b
% of CD4⁺ T cells
T_N
T_Eff
T_EM
T_CM
CTRL MG
c
% of antigen-exp CD4⁺ T cells expressing cytokine
GMCSF
IFNg
IL10
IL13
IL17A
IL2
IL21
TNF
IL6
IL9
IL22
IL3
IL4
CTRL MG
d
% of CD4⁺ T_EM cells expressing cytokine
GMCSF
IFNg
IL10
IL13
IL17A
IL2
IL21
TNF
IL6
IL9
IL22
IL3
IL4
CTRL MG
e
cytokine coexpression cluster 7
p = 0.0282
R² (adj.) = 0.336
% of CD4⁺ T_EM cells
relative Besinger score

Supplementary Figure 2. Cytokine polarization of circulatory Th cells is reduced in MG patients compared to healthy controls.

a: Biaxial plots showing the expression of CD45R0 and CCR7 in FlowSOM-generated naïve, effector and memory Th subsets.

b: Violin plots comparing the frequencies of memory Th cell clusters in healthy CTRLs and MG patients that did not receive immunomodulatory treatment.

c: Violin plots showing the frequency of expression of the 13 detected cytokines in all antigen-experienced $CD4^+$ T cells (T_{Eff}, T_{EM} and T_{CM}) from MG patients and healthy controls.

d: Violin plots showing the frequency of expression of the 13 detected cytokines in $CD4^+$ T_{EM} cells from MG patients and healthy controls.

e: Representative example of the correlation between the frequency of cytokine coexpression cluster 7 (c7) among $CD4^+$ T_{EM} and the modified quantitative MG score as a measure of disease activity.

Violin plots contain a bold horizontal line depicting the respective group mean. If not indicated, differences between experimental groups were statistically not significant ($p > 0.05$) using a nonparametric Mann-Whitney-Wilcoxon test with a false discovery correction according to the Benjamini-Hochberg approach. * = $p < 0.05$.

For correlation analysis statistical parameters were obtained by using a linear regression model. Shaded areas in **e** represent the 95% confidence interval.

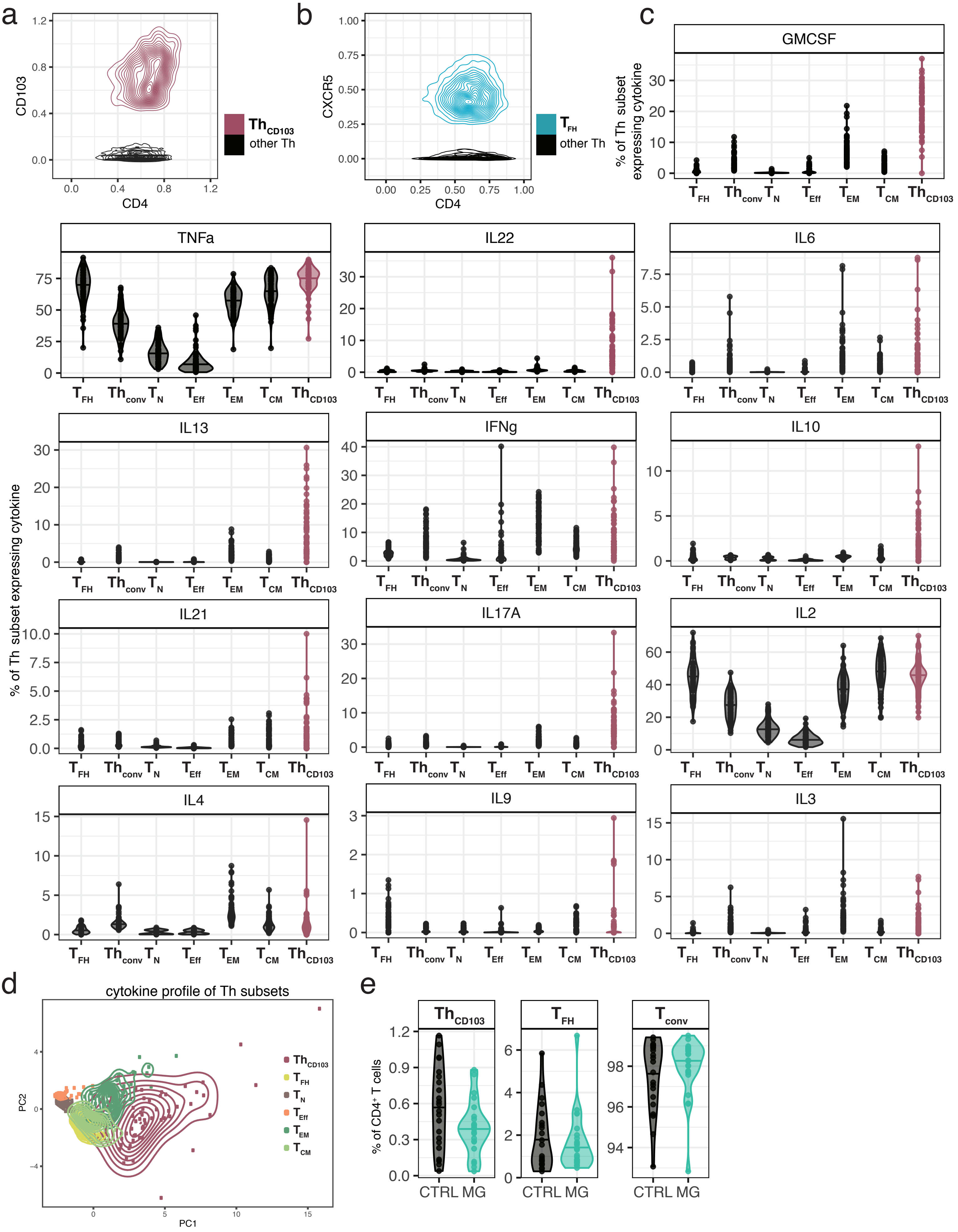

a
CD103
CD4
Th CD103
other Th
b
CXCR5
CD4
T FH
other Th
c
GMCSF
% of Th subset expressing cytokine
T FH
Th conv
T N
T Eff
T EM
T CM
Th CD103
TNFa
IL22
IL6
IL13
IFNg
IL10
IL21
IL17A
IL2
IL4
IL9
IL3
% of Th subset expressing cytokine
d
cytokine profile of Th subsets
PC2
PC1
Th CD103
T FH
T N
T Eff
T EM
T CM
e
Th CD103
T FH
T conv
% of CD4+ T cells
CTRL MG
CTRL MG
CTRL MG

Supplementary Figure 3. Circulatory Th_{CD103} cells represent potent cytokine producers

a and **b:** Biaxial plots showing the CD103 expression (**a**) and CXCR5 expression (**b**) of CD4-expressing Th_{CD103} and follicular Th (Th_{FH}) cells respectively in relation to the remaining Th compartment.

c: Violin plots comparing the frequency of expression of 13 different cytokines among the different Th subsets from all patients (CTRL and MG) in the cohort. Conventional Th cells (Th_{conv}) were further subdivided based on their memory profile into T_N, T_{Eff}, T_{EM} and T_{CM}.

d: Principle component analysis of the frequency of cytokine positivity for 13 analyzed cytokines for the indicated Th subpopulations for all patients (CTRL and MG) enrolled in the study.

e: Frequency of Th_{CD103}, follicular Th (Th_{FH}) cells and conventional Th cells (T_{conv}) in the blood of healthy controls and MG patients that did not receive immunomodulatory treatment.

Violin plots contain a bold horizontal line depicting the respective group mean. If not indicated, differences between experimental groups were statistically not significant ($p > 0.05$) using a nonparametric Mann-Whitney-Wilcoxon test with a false discovery correction according to the Benjamini-Hochberg approach. * = $p < 0.05$.

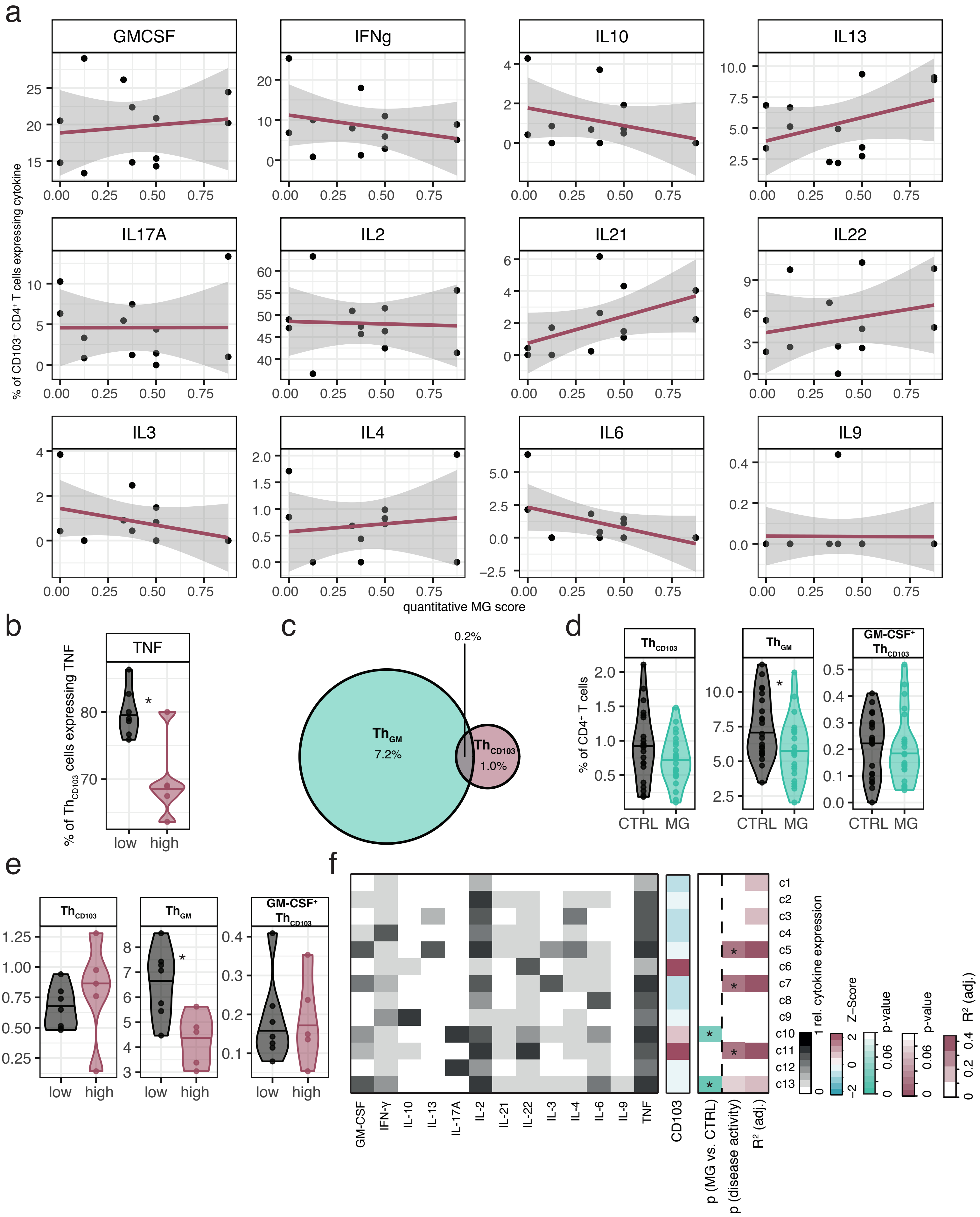
a
GMCSF
IFNg
IL10
IL13
IL17A
IL2
IL21
IL22
IL3
IL4
IL6
IL9
% of CD103⁺ CD4⁺ T cells expressing cytokine
quantitative MG score
b
TNF
% of Th CD103 cells expressing TNF
low
high
c
0.2%
Th GM
7.2%
Th CD103
1.0%
d
Th CD103
Th GM
GM-CSF⁺ Th CD103
% of CD4⁺ T cells
CTRL
MG
e
Th CD103
Th GM
GM-CSF⁺ Th CD103
% of CD4⁺ T cells
low
high
f
GM-CSF
IFN-γ
IL-10
IL-13
IL-17A
IL-2
IL-21
IL-22
IL-3
IL-4
IL-6
IL-9
TNF
CD103
p (MG vs. CTRL)
p (disease activity)
R² (adj.)
c1
c2
c3
c4
c5
c6
c7
c8
c9
c10
c11
c12
c13
rel. cytokine expression
Z-Score
p-value
p-value
R² (adj.)

Supplementary Figure 4. Th_{GM} and Th_{CD103} cells display minimal subset overlap

a: Correlation between the frequency of cytokine-producing Th_{CD103} cells and the modified quantitative MG score as a measure of clinical disease severity for newly-diagnosed patients neither receiving immunomodulatory nor symptomatic treatment.

b: Violin plot showing the frequency of TNF expression in Th_{CD103} cells in low and high disease severity newly-diagnosed treatment-naïve MG patients. Clinical disease severity was determined by the modified quantitative MG score, with low disease severity scoring < 0.5, and high disease severity ≥ 0.5.

c: Venn diagram showing the frequency of Th_{CD103} cells and Th_{GM} cells including the intersection of both subsets.

d and **e**: Violin plots showing the frequency of Th_{CD103} cells that do not express GM-CSF (left panel), Th_{GM} cells that do not express CD103 (middle panel) and GM-CSF-expressing Th_{CD103} cells (right panel) in the blood of healthy controls and MG patients that did not receive immunomodulatory treatment (**d**) and in low and high disease severity newly-diagnosed treatment-naïve MG patients (**e**).

f: CD103 expression in cytokine producing $CD4^+$ T_{EM} cell clusters as presented in **Fig. 2d**. Corresponding cytokine expression profiles (left box), column-normalized CD103 expression (middle box) as well as statistical parameters (right box) are displayed. Blue color indicates high significance (low p-value) for the comparison of treatment-naïve MG patients vs. CTRL, red color indicates high significance and high R^2 value respectively for the correlation with the continuous clinical disease severity.

Violin plots contain a bold horizontal line depicting the respective group mean. If not indicated, differences between experimental groups and linear correlations were statistically not significant ($p > 0.05$) using a linear model or a nonparametric Mann-Whitney-Wilcoxon test with a false discovery correction according to the Benjamini-Hochberg approach. * = $p < 0.05$.

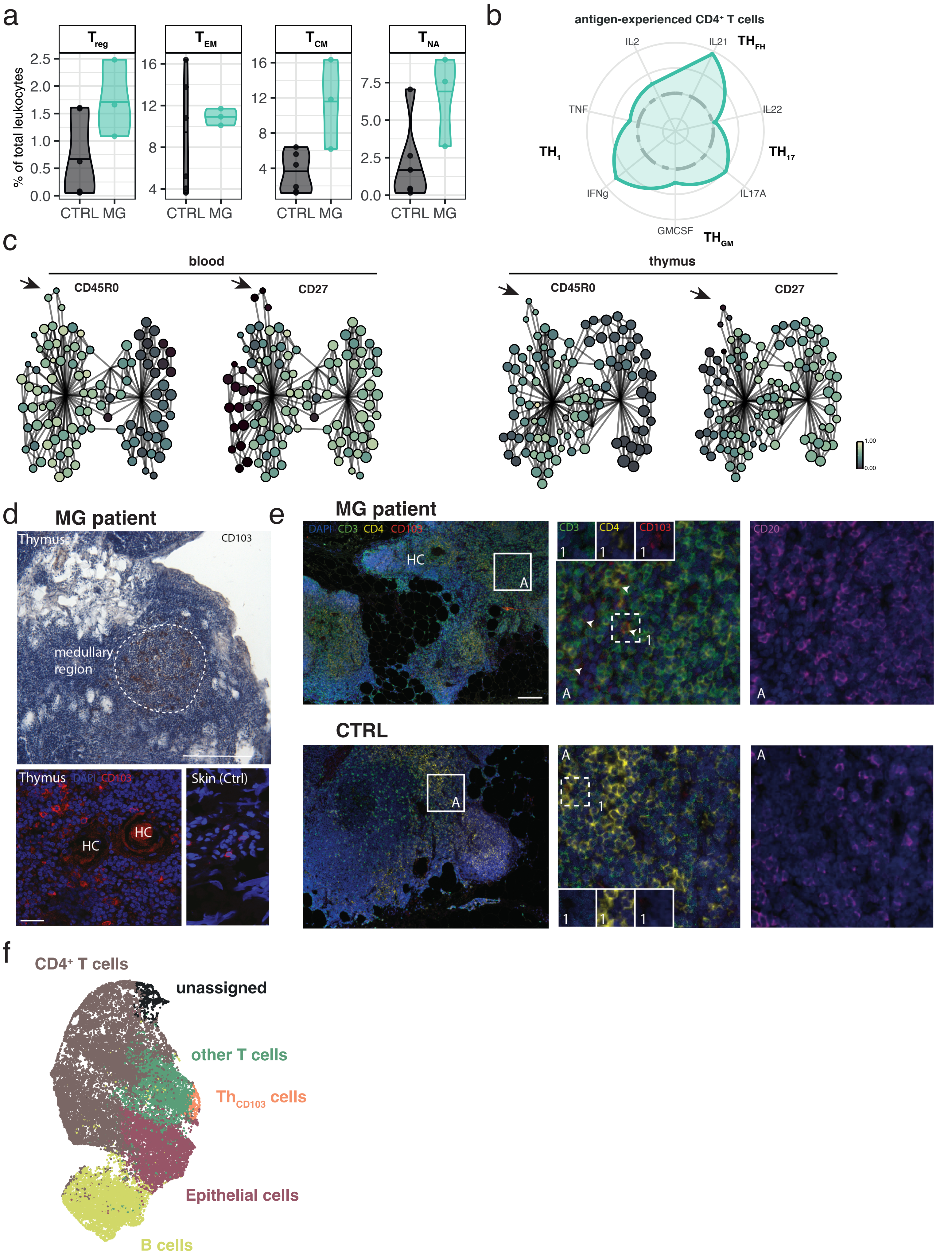

a
Treg
TEM
TCM
TNA
% of total leukocytes
CTRL MG
b
antigen-experienced CD4+ T cells
IL2
IL21
THFH
TNF
IL22
TH1
TH17
IFNg
IL17A
GMCSF
THGM
c
blood
thymus
CD45R0
CD27
1.00
0.00
d
MG patient
Thymus
CD103
medullary region
Thymus DAPI CD103
HC
Skin (Ctrl)
e
MG patient
DAPI CD3 CD4 CD103
CD3
CD4
CD103
CD20
CTRL
f
CD4+ T cells
unassigned
other T cells
ThCD103 cells
Epithelial cells
B cells

Supplementary Figure 5. Thymic Th_{CD103} cells are phenotypically related to peripheral Th_{CD103} cells and reside in medullary regions of MG patients.

a: Violin plots comparing the frequency of thymic Th subpopulations in MG patients and non-MG controls.

b: Radar plot representing the cytokine profile of antigen-experienced thymic $CD4^+$ T cells (excluding T_{NA}). The colored line indicates the Cohen's d effect size for each cytokine (MG vs. CTRL) as a deviation from the grey dashed reference line. Th cytokine profiles were manually annotated based on partially-overlapping key cytokines.

c: Scaffold of the blood and thymic Th cell compartment in MG patients. Color overlay depicts expression of CD45R0 and CD27.

d: Immunohistochemistry (top panel) and immunofluorescence labeling (bottom panel) of medullary thymic regions showing CD103 (red) and DAPI (blue). Representative images of two independent experiments (1 slide with 2 sections/patient each) are shown. MG patient: early onset, female MG patient without immunosuppressive therapy and thymus follicular hyperplasia. Scale bars: 200 µm (top panel) and 30 µm (bottom panel). HC = Hasall`s corpuscle.

e: Immunofluorescence labeling of medullary thymic regions showing CD3 (green), CD4 (yellow), CD103 (red), CD20 (magenta) and DAPI (blue). Samples from 13 MG patients and 6 non-MG controls were analyzed. Images of the single labels are enlargements of specified regions. Representative images of two independent experiments (1 slide with 2 sections/patient each) are shown. MG patient: early onset, female MG patient receiving low-dose glucocorticoids with lymphoid thymic hyperplasia. CTRL, male patient with incidental mass, normal thymic tissue and thymoma. Scale bar: 200 µm.

f: UMAP of 28 500 cells randomly sampled from the combined dataset of quantitative immunofluorescence labeling of thymic sections after cell segmentation. Color code indicates FlowSOM clustering and manual annotation according to lineage marker expression profiles presented in **Fig. 4f**.

Violin plots contain a bold horizontal line depicting the respective group mean. If not indicated, differences between experimental groups were statistically not significant ($p > 0.05$) using a nonparametric Mann-Whitney-Wilcoxon test with a false discovery correction according to the Benjamini-Hochberg approach.

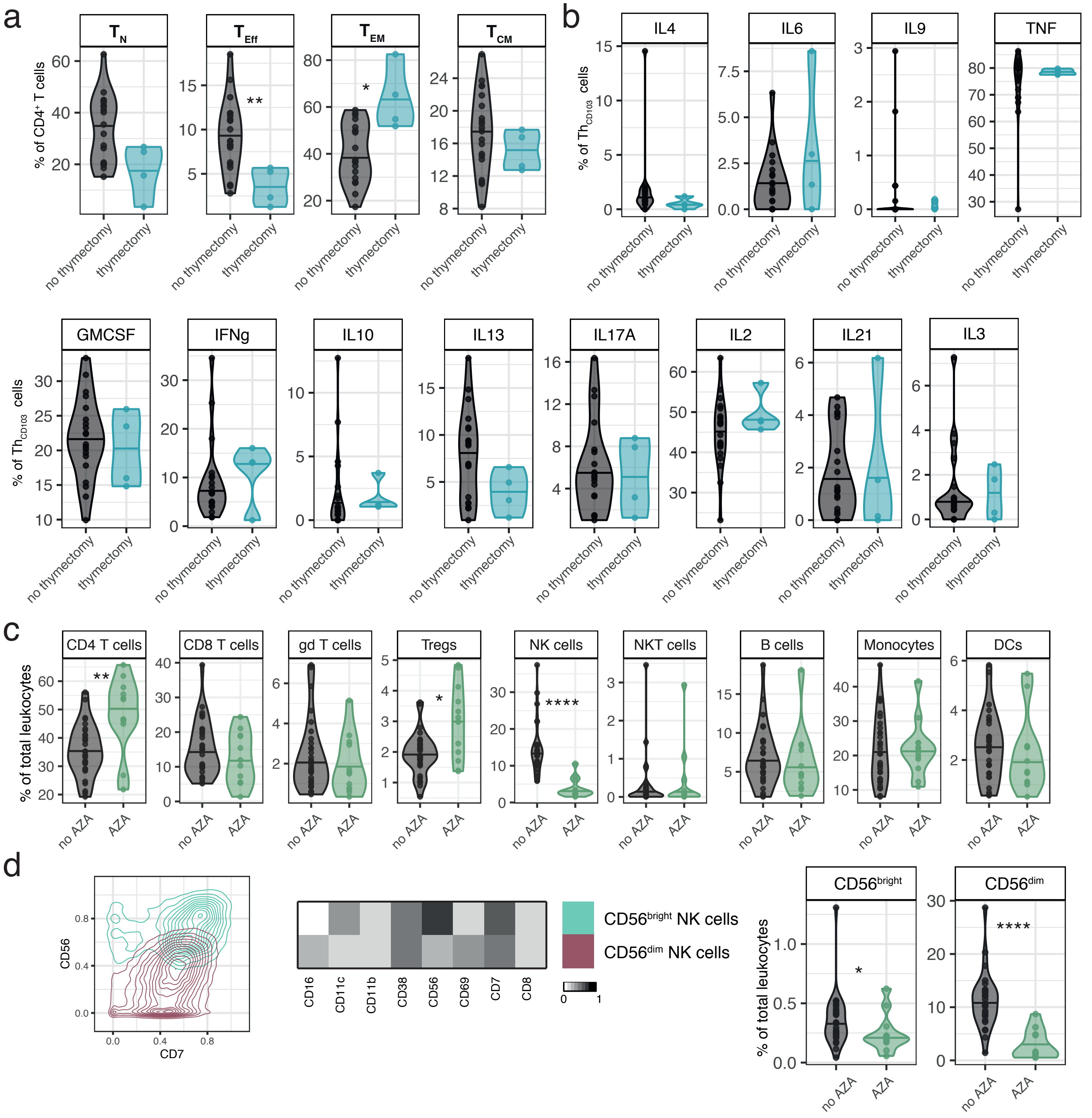

a
T N
T Eff
T EM
T CM
% of CD4+ T cells
no thymectomy
thymectomy
**
*
b
IL4
IL6
IL9
TNF
% of Th CD103 cells
GMCSF
IFNg
IL10
IL13
IL17A
IL2
IL21
IL3
c
CD4 T cells
CD8 T cells
gd T cells
Tregs
NK cells
NKT cells
B cells
Monocytes
DCs
% of total leukocytes
no AZA
AZA
****
d
CD56
CD7
CD16
CD11c
CD11b
CD38
CD56
CD69
CD7
CD8
CD56bright NK cells
CD56dim NK cells
CD56bright
CD56dim
% of total leukocytes

Supplementary Figure 6. Thymectomy and azathioprine modulate peripheral immune populations in MG patients.

a: Violin plots showing the frequency of memory Th cell subsets in the blood of thymectomized and non-thymectomized MG patients that did not receive immunomodulatory medication.

b: Violin plots showing the frequency of cytokine expression within Th_{CD103} cells, in the blood of MG patients with or without thymectomy that did not receive further immunomodulatory treatment.

c: Violin plots showing the frequency of major immune cell populations in the blood of azathioprine treated/untreated MG patients, as yielded by FlowSOM clustering.

d: Biaxial plot and heat map showing marker expression of FlowSOM-generated NK cell subsets. Violin plots depict frequencies of NK cell subsets in the blood of azathioprine treated vs untreated MG patients.

Violin plots contain a bold horizontal line depicting the respective group mean. If not indicated, differences between experimental groups were statistically not significant ($p > 0.05$) using a nonparametric Mann-Whitney-Wilcoxon test with a false discovery correction according to the Benjamini-Hochberg approach. * = $p < 0.05$; ** = $p < 0.01$; *** = $p < 0.001$; **** = $p < 0.0001$.

Isotope	Metal	Antigen	Clone	Supplier	Category	Panel
102	Pd	CD45	HI30	Biolegend	barcoding	1 + 2
104	Pd	CD45	HI30	Biolegend	barcoding	1 + 2
105	Pd	CD45	HI30	Biolegend	barcoding	1 + 2
106	Pd	CD45	HI30	Biolegend	barcoding	1 + 2
108	Pd	CD45	HI30	Biolegend	barcoding	1 + 2
110	Pd	CD45	HI30	Biolegend	barcoding	1 + 2
115	In	CD45	HI30	Biolegend	barcoding	1 + 2
141	Pr	VLA4	9F10	Fluidigm	surface	2
141	Pr	CCR6	G034E3	Fluidigm	surface	1
142	Nd	CD19	HIB19	Fluidigm	surface	1 + 2
143	Nd	CCR2	K036C2	Biolegend	surface	1 + 2
144	Nd	IL-4	MP4-25D2	Biolegend	ICS	2
144	Nd	CCR5	J418F1	Biolegend	surface	1
145	Nd	CD4	RPA-T4	Fluidigm	surface	1 + 2
146	Nd	CD8a	RPA-T8	Fluidigm	surface	1 + 2
147	Sm	CD11c	BU15	Biolegend	surface	1
147	Sm	IL-2	MQ1-17H12	Biolegend	ICS	2
148	Nd	CD16	3G8	Fluidigm	surface	1
148	Nd	IL-17A	BL168	Fluidigm	ICS	2
149	Sm	CCR4	205410	Fluidigm	surface	1
149	Sm	IL-3	BVD8-3G11	Biolegend	ICS	2
150	Nd	CD25	2A3	Biolegend	surface	1
150	Nd	IL-22	22URTI	Fluidigm	ICS	2
151	Eu	CD103	Ber-ACT8	Fluidigm	surface	2
151	Eu	CD38	HIT2	Biolegend	surface	1
152	Sm	TCRγδ	11F2	Fluidigm	surface	1 + 2
153	Eu	CD25	M-A251	Biolegend	surface	2
153	Eu	CD45RA	HI100	Fluidigm	surface	1
154	Sm	IL-6	MQ2-13A5	Biolegend	ICS	2
154	Sm	CD3	UCHT1	Fluidigm	surface	1
155	Gd	CD27	L127	Fluidigm	surface	1
155	Gd	IL-9	MH9A4	Biolegend	ICS	2
156	Gd	CD86	IT2.2	Fluidigm	surface	1
156	Gd	IL-13	JES10-5A2	Biolegend	ICS	2
158	Gd	CD10	HI10a	Fluidigm	surface	1
158	Gd	CCR4	205410	Fluidigm	surface	2
159	Tb	CD116	4H1	Biolegend	surface	1
159	Tb	GM-CSF	BVD2-21C11	Fluidigm	ICS	2
160	Gd	CD69	FN50	Biolegend	surface	1 + 2
161	Dy	CD20	2H7	Biolegend	surface	1 + 2

162	Dy	CD27	O323	Biolegend	surface	2
162	Dy	FoxP3	PCH101	Fluidigm	transcription factor	1
163	Dy	CD7	6B7	Biolegend	surface	1 + 2
164	Dy	ICAM-1	HA58	Biolegend	surface	1
164	Dy	CD45R0	UCHL1	Fluidigm	surface	2
165	Ho	CD127	A019D5	Fluidigm	surface	1
165	Ho	IFN-γ	B27	Fluidigm	ICS	2
166	Er	CD24	ML5	Fluidigm	surface	1
166	Er	IL-10	JES3-9D7	Fluidigm	ICS	2
167	Er	CCR7	G043H7	Fluidigm	surface	1 + 2
168	Er	CD154	24-31	Fluidigm	surface	1
168	Er	TNF	MAb11	Biolegend	ICS	2
169	Tm	CD33	WM53	Fluidigm	surface	1
169	Tm	CD45RA	HI100	Fluidigm	surface	2
170	Er	IgD	IA6-2	Biolegend	surface	1
170	Er	CD3	UCHT-1	Fluidigm	surface	2
171	Yb	CD138	MI15	Biolegend	surface	1 (second run)
171	Yb	CD22	HIB22	Biolegend	surface	1 (first run)
171	Yb	CXCR5	51505	Fluidigm	surface	2
172	Yb	IgM	MHM-88	Fluidigm	surface	1
172	Yb	IL-21	3A3-N2	Fluidigm	ICS	2
173	Yb	CD56	NCAM16.2	Biolegend	surface	1
173	Yb	CXCR4	12G5	Fluidigm	surface	2
174	Yb	PD-1	EH12.2H7	Fluidigm	surface	1 (second run) + 2 (first run)
174	Yb	HLA-DR	L243	Fluidigm	surface	2 (second run) + 1 (first run)
175	Lu	CD14	M5E2	Fluidigm	surface	1 + 2
176	Yb	CD5	UCHT2	Biolegend	surface	1
176	Yb	CD56	NCAM16.2	Biolegend	surface	2
191	Ir	DNA1	NA	Fluidigm	cell discrimination	1 + 2
193	Ir	DNA2	NA	Fluidigm	cell discrimination	1 + 2

195	Pt	Live/Dead	NA	Sigma	cell discrimination	1 + 2
209	Bi	CD16	3G8	Fluidigm	surface	2
209	Bi	CD11b	ICRF44	Fluidigm	surface	1

Supplementary Table 1. Heavy metal labeled antibodies used in the mass cytometry experiments.

Metal tagged antibodies were either purchased preconjugated (Fluidigm) or conjugated using the MaxPar X8 polymer (for other suppliers). NA = non-applicable; ICS = intracellular cytokine staining.

Fluorochrome	Antigen	Clone	Supplier	Category	Panel
Zombie UV	L/D	NA	Biolegend	cell discrimination	1 + 2
BUV395	CD45	HI-30	BD	surface	1
BUV661	HLA-DR	3G8	BD	surface	1
BUV563	CD25	2A3	BD	surface	1
BUV615	CD45RO	UCHL1	BD	surface	1
BUV737	CD19	SJ25C1	BD	surface	1
BUV805	CD3	UCHT1	BD	surface	1
BV570	CD8	RPA-T8	Biolegend	surface	1
BV421	PD1	EH12.2H7	Biolegend	surface	1
BV650	CD27	O323	Biolegend	surface	1
BV605	CD127	A019D5	Biolegend	surface	1 + 2
PE-Cy5.5	CD45RA	MEM-56	LifeTechnologies	surface	1
PE/Dazzle™ 594	CD56	5.1H11	Biolegend	surface	1
PE-Cy5	Pangd	IMMU510	Beckman Coulter	surface	1
AF488	CD4	RPA-T4	Biolegend	surface/ICS	1 + 2
Biotin	CD103	BER-ACT8	Biolegend	surface	1
BV711	IL-2	MQ1-17H12	Biolegend	ICS	1
BV785	TNF	Mab11	Biolegend	ICS	1
BB630	Strepavidin	NA	BD	ICS	1
BB790-P	CTLA4	BNI3	BD	ICS	1
PE	GM-CSF	BVD2-21C11	BD	ICS	1
PE-Cy7	IL-22	22URTI	ThermoScientific	ICS	1
A647	IL-21	3A3-N2.1	BD	ICS	1
A700	IFN-g	4S.B3	Biolegend	ICS	1
APC-C7	IL-17A	BL168	Biolegend	ICS	1
BUV395	CD103	Ber-ACT8	BD	surface	2
BUV496	CD19	SJ25C1	BD	surface	2
BUV661	CD11c	B-Ly6	BD	surface	2
BUV737	CD14	M5E2	BD	surface	2
BUV805	CD45R0	UCHL1	BD	surface	2
BV421	CD69	FN50	Biolegend	surface	2
BV480	CD27	L128	BD	surface	2
BV570	HLA-DR	L243	Biolegend	surface	2
BV650	IgD	IA6-2	BD	surface	2
BV711	CD45	HI-30	Biolegend	surface	2
BV785	CD38	HIT2	Biolegend	surface	2
PerCP-eFluor® 710	ICOS	ISA-3	ThermoScientific	surface	2

PE	CD25	M-A251	Biolegend	surface	2
PE/Dazzle™ 594	IgM	MHM-88	Biolegend	surface	2
PE-Cy5	CD5	L17F12	ThermoScientific	surface	2
PE-Cy7	CXCR5	MU5UBEE	ThermoScientific	surface	2
APC	CD3	UCHT1	Biolegend	surface	2
A647	IgG	*polyclonal*	Jackson	surface	2
AF700	CD8	3B5	LifeTechnologies	surface	2
APC-C7	CD56	HCD56	Biolegend	surface	2

Supplementary Table 2. Fluorochrome labeled antibodies used in the spectral flow cytometry experiments.

Fluorochrome labelled antibodies were purchased preconjugated. NA = non-applicable; ICS = intracellular cytokine staining.

Supplementary Data 1. Clinical and demographic characteristics of all MG and control patients enrolled in the mass cytometry study (separate file).

Disease duration was calculated based on first manifestation of disease symptoms. When no date for first manifestation of symptoms was available, the date of diagnosis was used instead. Cutoff for the differentiation between late and early onset was 50 years. MG = Myasthenia Gravis; CTRL = control; M = male; F = female; AChEI = acetylcholine esterase inhibitor; y = yes; n = no; MGFA = MG Foundation of American Classification; unk = unknown; NA = non-applicable

Supplementary Data 2. Clinical and demographic characterization of patients enrolled in the thymus spectral cytometry study (separate file).

Histology staging was performed by a pathologist independent of this study. MG = Myasthenia gravis; CTRL = control; m = male; f = female; y = yes; n = no; NA = non-applicable; MMF = mycophenolate mofetil; MTX = methotrexate.

Supplementary Data 3. Clinical and demographic characterization of patients enrolled in the thymus IHC study (separate file).

Histology staging was performed by a pathologist independent of this study. anti-AChR and -titin antibodies were measured before (or in the year of) thymectomy. CTRLs did

not show myasthenic symptoms during the follow-up period. MG = Myasthenia gravis; CTRL = control; m = male; f = female; y = yes; n = no; NA = non-applicable; unk = unknown; MMF = mycophenolate mofetil; MTX = methotrexate; AZA = azathioprine.
